# NH36 and F3 Antigen-Primed Dendritic Cells Show Preserved Migrating Capabilities and CCR7 Expression and F3 Is Effective in Immunotherapy of Visceral Leishmaniasis

**DOI:** 10.3389/fimmu.2018.00967

**Published:** 2018-05-07

**Authors:** Dirlei Nico, Fernanda Martins Almeida, Juliana Maria Motta, Fellipe Soares dos Santos Cardoso, Celio Geraldo Freire-de-Lima, Leonardo Freire-de-Lima, Paula Melo de Luca, Ana Maria Blanco Martinez, Alexandre Morrot, Clarisa Beatriz Palatnik-de-Sousa

**Affiliations:** ^1^Departamento de Microbiologia Geral, Instituto de Microbiologia Paulo de Góes, Universidade Federal do Rio de Janeiro, Rio de Janeiro, Brazil; ^2^Programa de Pós Graduação em Anatomia Patológica, HUCFF, Universidade Federal do Rio de Janeiro, Rio de Janeiro, Brazil; ^3^Programa de Graduação de Histologia, Instituto de Ciências Biomédicas, Universidade Federal do Rio de Janeiro, Rio de Janeiro, Brazil; ^4^Programa de Glicobiologia, Instituto de Bioquímica Médica Leopoldo De Meis, Universidade Federal do Rio de Janeiro, Rio de Janeiro, Brazil; ^5^Programa de Imunobiologia, Instituto de Biofísica Carlos Chagas Filho, Universidade Federal do Rio de Janeiro, Rio de Janeiro, Brazil; ^6^Programa de Medicina Regenerativa, Instituto de Biofísica Carlos Chagas Filho, Universidade Federal do Rio de Janeiro, Rio de Janeiro, Brazil; ^7^Laboratório de Imunoparasitologia, Instituto Oswaldo Cruz (IOC), Rio de Janeiro, Brazil; ^8^Centro de Pesquisas em Tuberculose, Faculdade de Medicina, Universidade Federal do Rio de Janeiro, Rio de Janeiro, Brazil; ^9^Instituto Nacional de Ciência e Tecnologia de Investigação em Imunologia, São Paulo, Brazil

**Keywords:** visceral leishmaniasis, dendritic cells defective migration, CCR7 expression, nucleoside hydrolase, NH36, F3 domain, *Leishmania donovani*, *Leishmania infantum chagasi*

## Abstract

Physical contact between dendritic cells (DCs) and T cell lymphocytes is necessary to trigger the immune cell response. CCL19 and CCL21 chemokines bind to the CCR7 receptor of mature DCs, and of T cells and regulate DCs migration to the white pulp (wp) of the spleen, where they encounter lymphocytes. In visceral leishmaniasis (VL), cellular immunosuppression is mediated by impaired DC migration due to the decreased chemokine secretion by endothelium and to the reduced DCs CCR7 expression. The *Leishmania (L.) donovani* nucleoside hydrolase NH36 and its C-terminal domain, the F3 peptide are prominent antigens in the generation of preventive immunity to VL. We assessed whether these vaccines could prevent the migrating defect of DCs by restoring the expression of CCR7 receptors. C57Bl6 mice were vaccinated with NH36 and F3 and challenged with *L. (L.) infantum chagasi*. The F3 vaccine induced a 100% of survival and a long-lasting immune protection with an earlier CD4^+^Th1 response, with secretion of higher IFN-γ and TNF-α/IL-10 ratios, and higher frequencies of CD4^+^ T cells secreting IL-2^+^, TNF-α^+^, or IFN-γ^+^, or a combination of two or the three cytokines (IL-2^+^TNF-α^+^IFN-γ^+^). The CD8^+^ T cell response was promoted earlier by the NH36-vaccine, and later by the F3-vaccine. Maximal number of F3-primed DCs migrated *in vitro* in response to CCL19 and showed a high expression of CCR7 receptors (26.06%). Anti-CCR7 antibody treatment inhibited DCs migration *in vitro* (90%) and increased parasite load *in vivo*. When transferred into 28-day-infected mice, only 8% of DCs from infected, 59% of DCs from NH36-vaccinated, and 84% of DCs from F3-vaccinated mice migrated to the wp. Consequently, immunotherapy of infected mice with F3-primed DCs only, promoted increases in corporal weight and reductions of spleen and liver parasite loads and relative weights. Our findings indicate that vaccination with F3-vaccine preserves the maturation, migration properties and CCR7 expression of DCs, which are essential processes for the generation of cell-mediated immunity. The F3 vaccine is more potent in reversing the migration defect that occurs in VL and, therefore, more efficient in immunotherapy of VL.

## Introduction

Leishmaniasis is still considered one of the most neglected diseases in the world (1). Approximately, 350 million people are at risk, and about two million new cases are registered annually (2). More than 20 *Leishmania* species are involved in the transmission of Leishmaniasis. The parasites are transferred to humans by hematophagous phlebotomine sandflies (3). *Leishmania (L.) donovani, L. (L.) infantum*, and *Leishmania (L.) infantum chagasi* are the agents of visceral leishmaniasis (VL). While the disease is anthroponotic in India and East Africa, in the Americas, North Africa, Asia, and the Mediterranean, VL is a canid zoonosis (4). Clinical signs of human VL, include fever, malaise, anorexia, cachexia, hypergammaglobulinemia, hepato- and splenomegaly, anemia, and progressive suppression of the cellular immune response. Currently, the annual incidence reaches 400 thousands cases and 30 thousands deaths worldwide (5). Ninety percent of VL cases are registered in India, Ethiopia, South Sudan, Bangladesh, Sudan, and Brazil. Although the VL control programs in South-East Asia are reducing the human incidence of the disease, and the number of VL cases declined in Bangladesh, India, and Nepal (3), recurrent outbreaks of VL in and Sudan, Kenya, Ethiopia, and South Sudan are raising concern.

The development of the cellular immune response requires that T cell lymphocytes make contact with dendritic cells (DCs) in the spleen and lymph nodes (6, 7). When the spleen is chronically infected with *Leishmania* parasites, the structural design of the B cell follicles and the marginal zone (MZ) are disrupted (8–10). This disorder determines an insufficient antigen presentation to T cells. In fact, splenic T cells and DCs move from the MZ to the periarteriolar lymphoid sheath (PALS), where there are increased concentrations of chemokines (11). CCL19 and CCL21 chemokines produced by endothelium venules (12) bind to CCR7 receptor. These chemokines are attractants to T cell naïve lymphocytes, mature DCs, and a subset of memory T cells (12, 13). Therefore, although the number of splenic DCs increases after *L. (L.) donovani* infection (11), they fail to migrate to PALS, due to the reduced chemokine secretion by PALS, and to the inhibition of CCR7 expression on DCs (11). This spatial separation of T lymphocytes and DCs impedes their physical contact and is one of the reasons of the suppression of cellular immune response in VL (11).

Confirming the results obtained by Ato et al. (11) who studied the *L. (L.) donovani* infections, we recently demonstrated that mice chronically infected with *L. (L.) infantum chagasi* also show an increased spleen relative weight, correlated to a DCs hyperplasia, and to an increased spleen parasite load (14). In contrast, mice vaccinated with the nucleoside hydrolase (NH36) recombinant antigen, or with its C-terminal moiety (F3) and saponin, showed a strong reduction in spleen parasite load and prevented the hyperplasia of spleen DCs and an increase in spleen relative weight (14).

NH36 is a promising vaccine antigen, which protects mice and dogs from VL infection (15–18). NH36 domains and epitopes are recognized by PBMC of subclinical and cured human patients from Brazil (19) and from Spain (20). F3 holds the most important NH36-epitopes for antibodies and MHC class II receptors of mice and induces a CD4^+^ T-cell-mediated protection against VL, correlated with an enhanced TNF-α and strong decrease of IL-10 secretion (15, 21).

No human vaccine against VL has been licensed until now and chemotherapy shows toxicity and failure issues (22, 23). Leishmaniasis remains as one of the main parasitic diseases of major impact on humanity and the search for isolated, combined, or alternative therapies that are safe, effective, and easily administered for the treatment of VL remains a promising target study. In this investigation, we take a step further in the study of the efficacy of the NH36 and F3 vaccines against murine VL and demonstrate that, besides preventing the increase of the numbers of DCs, they also prevent the migration dysfunction of DCs by restoring their CCR7 receptor expression. Additionally, we show that immunotherapy of infected mice with DCs derived from animals vaccinated with the F3 domain is a potential tool to assist in the treatment of the VL.

## Materials and Methods

### Ethical Statement and Biosafety Measures

Protocol design of the experiments was approved by the Comissão de Ética no Uso de Animais of the Universidade Federal do Rio de Janeiro (CEUA protocol IMPPG040-07/16), in agreement with Brazilian laws for animal safety and the guidelines of National Institute of Health (15). Animals were maintained at the Instituto de Microbiologia Paulo de Góes, which is part of Universidade Federal do Rio de Janeiro (UFRJ) facilities, given water and food *ad libitum*, with a 12 h light/dark cycles and controlled temperature (15). We aimed to reduce any animal suffering to a minimum.

In this investigation, we worked with genetically modified *Escherichia coli* BL21 and DH5 strains cloned with the pET28b plasmid expressing the NH36 and F3 recombinant proteins. These bacterial clones used in this investigation are considered OGM risk level 1 (CQB 0108/99 IMPG-UFRJ) because they are not associated with disease in adults. We also manipulated non-genetically modified *Leishmania* parasites for which the CTNBio biosafety level is 2. Following the Brazilian Ministry of Health Biosafety regulations for Biomedical and Microbiology laboratories we performed the following biosafety measures: use of apron, gloves, masks, and protective glasses, decontamination of infected biological material and animal cases before washing and limited access to, security block and signalizing of the risk area. Additionally, we used laminar flow, aseptic chambers, automatic pipetters, and reservoirs with sterilizing solutions. On the day of the experiments with the bacterial clones, no other manipulation of microorganisms occurred. All the non-disposable material and surgery tools are autoclaved. A separated manipulation room with an ultraviolet and exhaust chamber was used when necessary. Carcasses were incinerated.

### Recombinant NH36 Antigen and F3 Domain

NH36 is composed of 314 amino acids. The NH36 sequence is deposited in SWISS-PROT (accession code Q8WQX2), EMBL (AY007193), GenBank™ and DDJB (AAG02281.1) data bases (15, 20). F3 is composed of the amino acids 199–314 of NH36. The sequences of the NH36 and F3 were cloned in the pET28b plasmid system, expressed in *E. coli* Bl21DE3 cells and purified by affinity chromatography (Ni-NTA, Qiagen), as described before (15, 20). The presence or absence of LPS was confirmed using the LAL QCL-1000 kit (Lonza). The levels of LPS were lower than the sensitivity range of the Limulus amebocyte lysate test, which is 0.1–1.0 EU/ml. Therefore, there was no need for endotoxin removal.

### Vaccination and Challenge With *L. (L.) infantum chagasi*

Eight-week-old C57BL/6 female mice, obtained from the Fiocruz-Cecal facilities (Rio de Janeiro, Brazil) were vaccinated subcutaneously, with three doses of 100 µg of NH36 or F3 formulated with 100 µg of saponin (SIGMA), at weekly intervals (15). Control mice received only saline. On week 4, mice were challenged with an intravenous injection in the tail vain of 3 × 10^7^ *L. (L.) infantum chagasi* amastigotes (strain IOC-L 3324) isolated from infected hamsters’ as described before (24, 25). On day 28 after infection, euthanasia was performed and the variation in total body weight and relative liver/body weight were recorded (15). The parasite load in the livers was determined by microscopic observation of Giemsa-stained impression smears. The parasite burden was assessed as LDU values (number of amastigotes per 1,000 of organ cell nuclei/mg of organ weight) (11, 15, 16, 18, 25). Alternatively, the parasite burden was assessed by a limiting dilution assay (LDA) of the aseptically removed liver fragments suspended in a 1/5 serial dilution in Schneider’s medium, and incubated at 26°C. Promastigotes present in the last well containing visible parasites were quantified in a hemocytometer (21). Two-independent experiments were performed with *n* = 7 mice per treatment in each treatment.

### Assessment of the Intradermal Response to Leishmanial Antigen (IDR)

IDR against *L. (L.) donovani* lysate was measured in the footpads on day 7 after immunization and on day 25 after challenge (15, 26). The right hind footpads were injected intradermally with 10^7^ freeze–thawed *L. (L.) donovani* promastigotes at the stationary phase (15, 26–28). Before, and at 24 and 48 h after injection the swelling was assessed with a Mitutoyo apparatus. Each animal received only 0.1 ml saline in the left hind footpad as control. Values of the saline control were subtracted from the reaction due to *Leishmania* antigen, at each measurement (15, 26). Two-independent experiments were performed with *n* = 6–7 C57BL/6 mice per treatment in each experiment.

### DCs Isolation and Migration Tests *In Vitro* and *In Vivo*

Dendritic cells was isolated from spleens of vaccinated mice, 28 days after challenge, following the instructions of the magnetic beads manufacturer (Miltenyi Biotec, USA). Briefly, spleens were collected, shredded, and incubated with collagenase, at 1 mg/ml, and DNAse (Sigma) at 20 µg/ml concentration (29), respectively, for 20 min, at room temperature (Figure S1 in Supplementary Material). After that, the spleens were macerated and the splenocytes passed through a cell mesh. Also an ACK solution was used for erythrocyte lysis. Magnetic microbeads conjugated with anti-mouse CD11c were added to the preparation and then incubated for an additional 30 min. After that, the DCs were purified using MACS magnetic columns (Miltenyi Biotec, Germany) (11) and labeled with anti-mouse CD11c antibody conjugated to fluorescein isothiocyanate (FITC), clone N418 (eBiosciense, USA). Cells were additionally incubated with allophycocyanin (APC)-conjugated to anti-CCR7 (eBioscense) (two experiments with *n* = 5 mice per treatment for each experiment) (Figure S1 in Supplementary Material). The cytometry analysis was performed using a FACSCalibur apparatus (Becton Dickinson).

The *in vitro* migration assays used DCs purified from C57BL/6 naïve, *L. (L.) infantum chagasi* infected and NH36 or F3 vaccinated and infected mice. The DCs (5 × 10^5^ per well) were suspended in RPMI containing 1% fetal calf serum and placed into the upper chamber of Transwell inserts (5 µm pore size, Coaster, Corning). The inserts were further positioned in plates containing RPMI, with or without the addition of 100 ηM CCL19 (R&D Systems). After 2 h of incubation at 37°C, the cells in the lower wells of the inserts were collected and counted in a hemocytometer chamber. For the purity analysis, cells which migrated were stained with anti-mouse CD11c antibody, and analyzed by flow cytometry. Two-independent experiments were performed, each one of them with *n* = 5–6 mice per treatment. In order to prove that the DCs migration is due to the sensitivity of CCR7 receptors to the CCL19 chemokine gradient, blocking experiments were performed by incubating the DCs obtained from infected or F3sap-vaccinated mice with anti-CCR7 antibody (e Bioscience) at 1/50 dilution for 30 min at 4°C, before the migration assay in transwell plates, as described.

The migration assays *in vivo* used DCs purified from spleens of *L. (L.) infantum chagasi* infected and NH36 or F3-vaccinated, or from infected mice. Isolation and purification of DCs were performed using magnetic beads as described for the *in vitro* assays (Figure S1 in Supplementary Material). Additionally, the DCs were stained with two drops/ml (6 µg/ml) of Hoescht 33342 (Life Technologies), for 20 min at 37°C, protected from light. After that, 10^6^ labeled DCs were injected intravenously into the infected receptors, on day 28 after infection, as described by Ato et al. (11). Spleens of receptors were removed 24 h after the DCs transfer and immersed in paraformaldehyde, followed by cryoprotection in an increasing sucrose gradient (10, 20, and 30%). Cryostat sections (16 µm) were performed and mounted in Fluoromount and observed under a confocal microscope (LeicaTCS SP5). Sections from three mice of each treatment were chosen randomly and analyzed for the presence of labeled DCs in the white pulp (wp) or in the red pulp (rp) or in the MZ. Approximately 1,000 stained DCs were recorded and the differences between the proportions detected in the wp, and the rp, were analyzed by the Fischer exact test for comparison of proportions (https://www.graphpad.com/quickcalcs/contingency2/). Two-independent experiments were performed, each one of them with *n* = 3 receptor mice per treatment.

### DCs Immunotherapy

Splenic DCs from *L. (L.) infantum chagasi*-infected mice, and from NH36 or F3-vaccinated and challenged mice, were purified on day 28 after infection. The total amount of purified DCs obtained from the spleens from the different experimental groups varied from 1 to 2.6 × 10^6^. For the immunotherapy experiments, 10^6^ DCs of each group of mice were injected intravenously into *L. (L.) infantum chagasi*-infected C57BL/6 recipients on day 28 post infection. The parasite loads in spleen and livers, the spleen and liver/corporal relative weights, and the variation in body weights were recorded in the recipient mice 7 days later by LDU. Two-independent experiments were performed with *n* = 5 mice per treatment in each experiment. As a control, blocking experiments to prove that migration of DC is critical for the protection in mice were performed using DCs obtained from normal, infected, and NH36, and F3-vaccinated and infected mice, that were pre-incubated with anti-CCR7 antibody and further transferred to infected receptors.

### Cytokine-Secretion Assay in Vaccinated Mice

Spleens were aseptically removed and splenocyte suspensions were prepared as described before (21). Purified DCs were also obtained. Cells were distributed into 96-well plate (10^6^ cell/well) and incubated with 5 µg/ml NH36 or lysate of stationary phase *L. (L.) infantum chagasi* promastigotes, or with no addition for 72 h *in vitro*, at 37°C with 5% CO_2_. After that, supernatants were harvested and assayed using the Mouse IFN gamma, TNF-α, and IL10 ELISA Ready-SET-Go! (e-Biosciences, USA). The sensitivity of the assay was established with a range of 10–1,000 pg/ml for TNF-α, 100–1,000 pg/ml for, and of 15–2,000 pg/ml for IFN-γ. Reactions were developed using biotinylated anti-cytokine antibodies, streptavidin (SAv-HRP) enzymatic reagent, and TMB (Zymed, USA). Absorbances were monitored in a BioRad ELISA reader at 655 ηm. Two-independent experiments (*n* = 5 mice per treatment in each experiment) were performed.

### Intracellular Cytokine Staining in Vaccinated Mice

The analysis of the T cell response used splenocytes stimulated *in vitro* with 5 µg/ml NH36 or lysate of stationary phase *L. (L.) infantum chagasi* promastigotes or with no addition for 24 h at 37°C with 5% CO_2_, as described (21), and with incubation with Brefeldin A (SIGMA). After that, the splenocytes were stained with rat anti-mouse-CD4FITC (clone GK1.5) and -CD8FITC (clone 53–6.7) monoclonal antibodies (eBioscience), fixed, washed, treated with 0.5% saponin-FACS buffer, and stained with IFN-γAPC, IL-2-PerCP-Cy5.5, and TNF-αPE monoclonal antibodies (eBioscience). A total of 100,000 events were acquired. Gating for CD4^+^ ad CD8^+^ T cells was performed using a FACSCalibur apparatus (Becton Dickinson). Data analysis was performed with the Flow-jo program (Treestar, USA). Two-independent experiments were performed with *n* = 5 mice per treatment in each experiment.

### Statistical Analysis

Means were compared using the Kruskal–Wallis and Mann–Whitney non-parametrical tests. Correlation coefficients were established by the Pearson’s two-tailed correlation test (GraphPad Prism 6 software) as described (21). Also compared the survival distribution of individuals in infected controls and vaccinated and challenged mice were compared using the Log-rank (Mantel–Cox) and the Gehan–Breslow–Wilcoxon tests (GraphPad Prism 6 software).

## Results

### Vaccination With F3 and NH36 Prevents Dysfunctional DCs Migration and Decreased CCR7 Expression

In the present investigation, we aimed to assess whether DCs from vaccinated mice preserve their correct migrating capabilities in response to CCL19 chemokine, and if this fact is related to their normal sustained expression of the CCR7 receptor.

**Figure 1:**
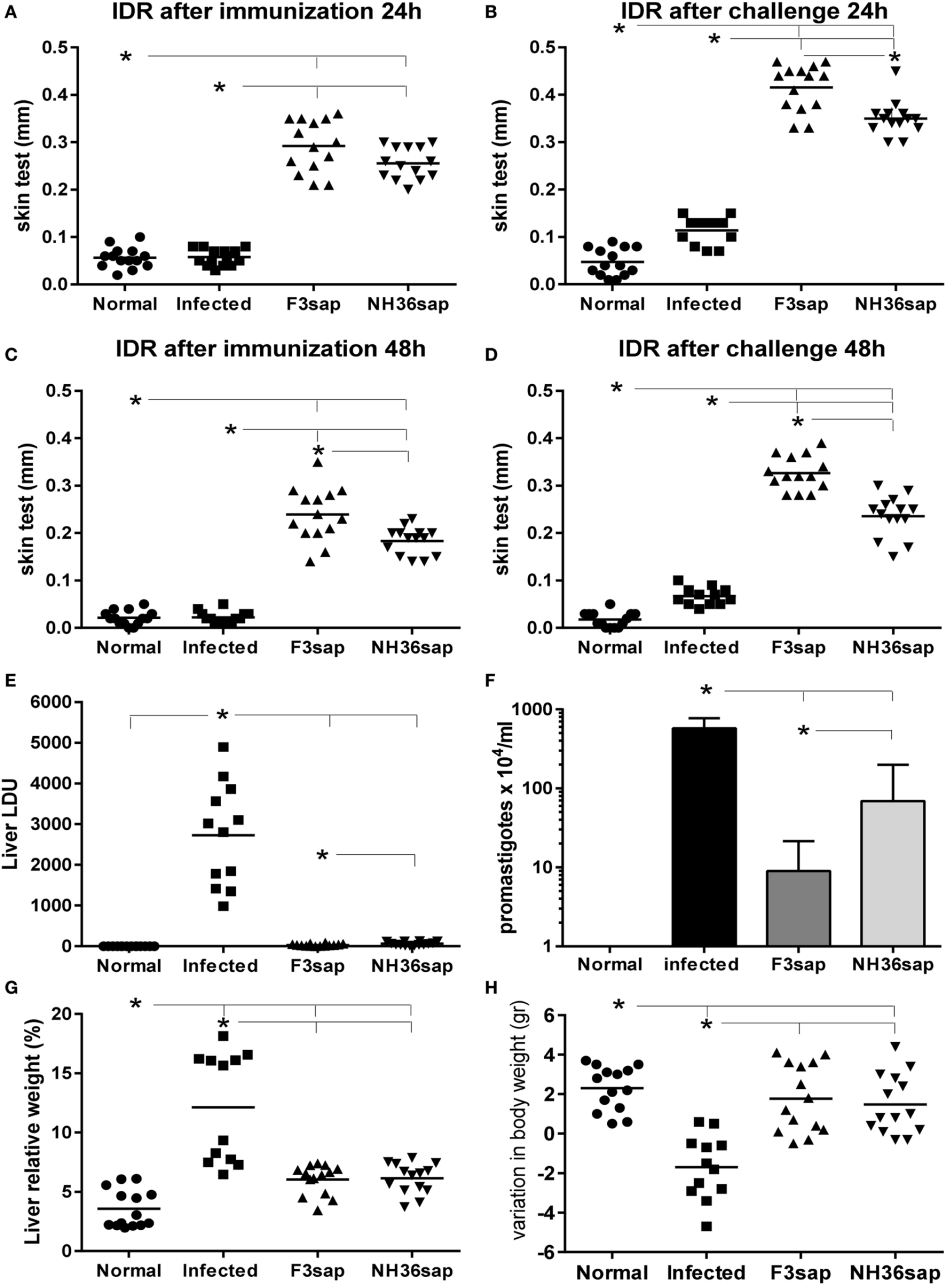
F3 and NH36-vaccinated dendritic cells donor mice showed enhanced IDR and reduced clinical and parasitological impact of infection. IDR to the leishmanial antigen was measured at 24 h **(A,B)** and 48 h **(C,D)** after complete immunization with the F3 (F3sap) and the NH36 (NH36sap) vaccines, and after immunization and challenge. Parasite load in liver is expressed as LDU values **(E)** and total promastigotes/ml, in *in vitro* cultures of livers as determined by the limiting dilution assay method **(F)**. Liver relative weight **(G)**, and gain in corporal weight in grams **(H)** were recorded after euthanasia. Results represent the individual values of two-independent experiments (*n* = 6–7 C57BL/6 mice per treatment for each experiment). Horizontal full lines represent the mean values. Asterisks and horizontal lines show significant differences between treatments as disclosed by Mann–Whitney non-parametrical test.

As a source of DCs we used normal, infected, and NH36 and F3-vaccinated and challenged mice. Initially, we confirmed that the vaccine-induced protection in the these mice by assessing the achievement of enhanced IDR responses, reduced liver parasite loads and liver relative weights, and sustained corporal weights (Figure 1). In fact, vaccination with both, the F3 and the NH36 vaccines, significantly increased the IDR after complete immunization (Figures 1A,C), and after challenge (Figures 1B,D) above the levels detected in normal control mice, at all tested times (24 and 48 h after injection). Remarkably, after infection, the F3 vaccine was 74 and 79% stronger than controls, at 24 and 48 h, respectively and 27% stronger (*p* < 0.0001) than the NH36 vaccine (Figures 1B,D).

IDR values correlated negatively with the liver/body relative weight after immunization (*p* < 0.0001, *R* = −0.5768, *R*^2^ = 0.3327 at 24 h and *p* < 0.0001, *R* = −0.5833, *R*^2^ = 0.3402 at 48 h after injection) and after challenge (*p* < 0.0001, *R* = −0.5989, *R*^2^ = 0.3587 at 24 h and *p* < 0.0001, *R* = −0. 6334, *R*^2^ = 0.4012 at 48 h after injection).

Notably, both vaccines strongly reduced the liver LDU values compared to those of infected mice (*p* < 0.0001) (Figure 1E). Compared to infected controls, F3 vaccine produced a 98.8% reduction, and the NH36 vaccine, a 97.6% of reduction of the parasite load (*p* = 0.0318) (Figure 1E). F3 vaccine reduced the LDUs values by 50% in comparison to the NH36 vaccine. The reduction of the parasite load determined by both vaccines was also confirmed by the LDA method (Figure 1F). The F3 vaccine induced 98% (*p* < 0.079), and the NH36 vaccine only 88% reduction (*p* < 0.0079) (*p* < 0.05) of the number of promastigote in liver culture. Additionally, the F3 vaccine reduced the promastigotes counts by 87% (*p* < 0.05) in comparison to the NH36 vaccine (Figure 1F). The LDU and LDA values for the liver parasite loads were positively correlated (*p* < 0.026, *R* = 0.496, *R*^2^ = 0.2464).

The increases in IDR after infection were strong correlates of protection regarding the liver LDU values. In fact, LDU values were negatively correlated with IDR after immunization, at 24 h (*p* < 0.0001, *R* = –0.7875, *R*^2^ = 0.6202) and at 48 h (*p* < 0.0001, *R* = −0.7674, *R*^2^ = 0.5890) and after challenge, at 24 h (*p* < 0.0001, *R* = −0.7796, *R*^2^ = 0.6077) and at 48 h (*p* < 0.0001, *R* = −0.7543, *R*^2^ = 0.5690).

Increases in liver/body relative weight were also significantly higher in infected control animals than in normal uninfected controls and in F3 and NH36-vaccinated and challenged mice (*p* < 0.0001 for all comparisons) (Figure 1G). In addition, only infected controls lose corporal weight (Figure 1H) in comparison to normal, F3 and NH36-vaccinated mice (*p* < 0.0001 for all comparisons). Vaccinated and normal mice showed similar gain in corporal weight. Besides, IDR was positively correlated to corporal weight gain after immunization (*p* = 0.0009, *R* = 0.5055, *R*^2^ = 0.2556, at 24 h and *P* = 0.0002, *R* = 0.5504, *R*^2^ = 0.3030, at 48 h after injection) and after challenge (*p* < 0.0001, *R* = 0.5888, *R*^2^ = 0.3467, at 24 h and *p* < 0.0001, *R* = 0.6532, *R*^2^ = 0.4267, at 48 h after injection).

**Figure 2:**
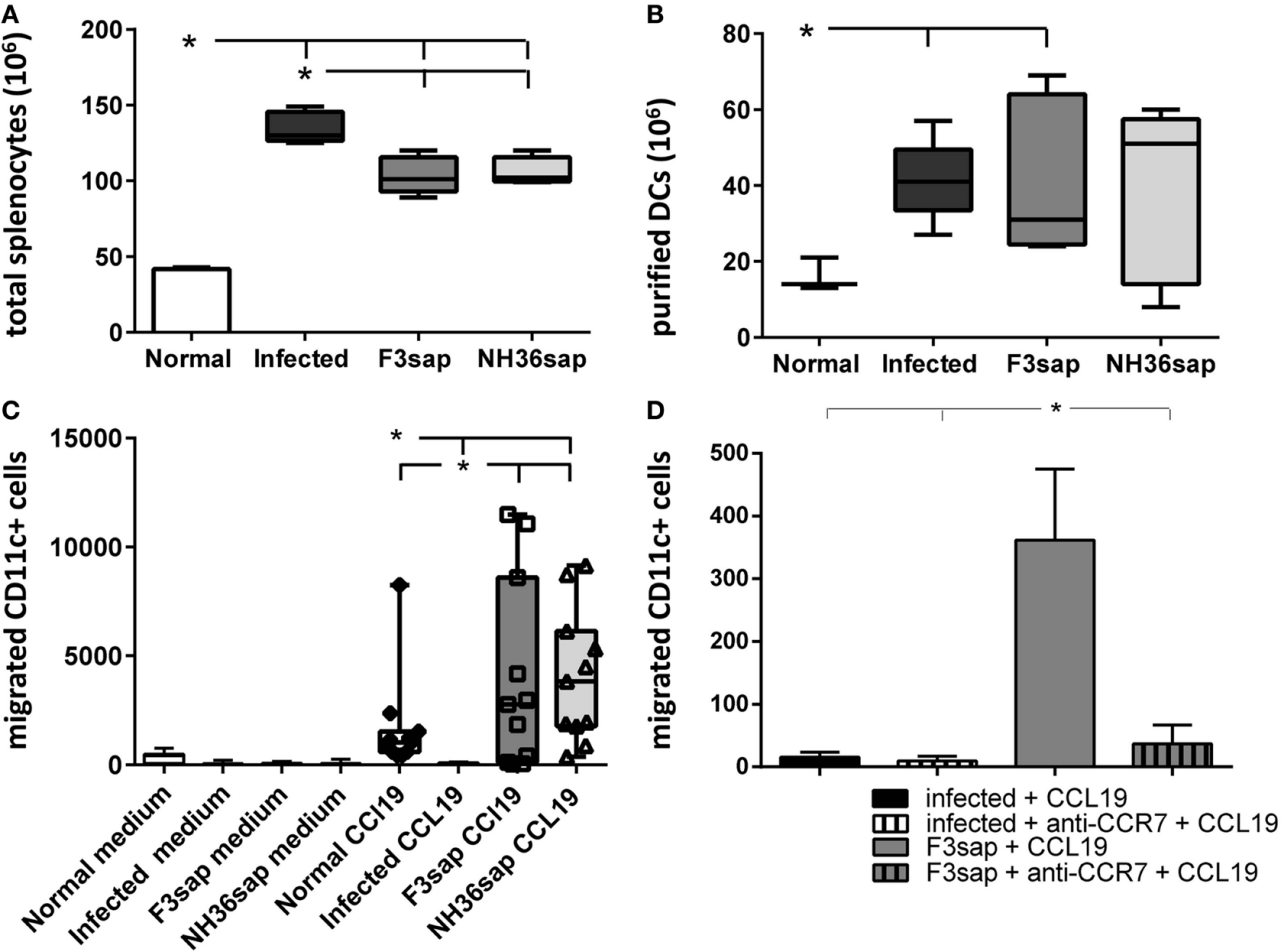
F3- and NH36-primed dendritic cells (DCs) show enhanced migration response to CCL19 chemokine. Splenic DCs were purified from, normal, infected and F3, and NH36-vaccinated and *Leishmania infantum chagasi*-challenged mice. DCs were isolated at day 28 after challenge. The whiskers show the maximal and minimum values, and the top and bottom of the bars show respectively, the 75th and 25th percentiles. The 75th percentile is the value below which 75% of observations in a group of observations fall. **(A)** Total splenocytes. **(B)** Number of DCS purified by magnetic beads-affinity chromatography. **(C)** The chemotactic responses to 100 ηM CCL19 were assessed using a transwell system. After 2 h of incubation, migrated DCs were collected, stained using fluorescein isothiocyanate-conjugated anti-CD11c antibody, and counted by flow cytometry in a FacsCalibur apparatus (Becton Dickinson). In the boxes, the individual results for migrating DCs counts of normal mice (diamonds), F3-vaccinated (squares), and NH36-vaccinated mice (triangles). **(D)** Number of DCs from infected or F3-vaccinated and challenged mice, pre-incubated with anti-CCR7 antibody or no addition, which migrated toward CCL19. Results represent two-independent experiments (*n* = 5–6, mice per treatment in each experiment). Asterisks and horizontal lines show significant differences between treatments as disclosed by Mann–Whitney non-parametrical test.

Once the generation of a protective response was confirmed, we further studied the migrating capabilities of DCs obtained from these vaccinated, naïve controls, and infected mice on day 28 after challenge. In Figure 2, we represent the results as boxes and whiskers. The whiskers show the maximal and minimum values and the top and bottom of the bars show, respectively, the 75th and 25th percentiles. The 75th percentile is the value below which 75% of observations in a group of observations fall. Figure 2A shows that the number of total splenocytes isolated was, as expected, higher in infected mice than in the two vaccinated groups. FACS cytometry analysis of the affinity chromatography purified CD11c^+^ DCs preparations disclosed 90–100% rates of purity. We further observed that the number of DCs purified from total splenocytes was higher in infected and F3-vaccinated, than in normal mice (Figure 2B).

Additionally, none of the DCs migrated toward the medium containing no additions (Figure 2C). In contrast, while DCs of normal controls and of F3 and NH36-vaccinated mice migrated toward the CCL19 gradient, DCs of infected animals did not (*p* < 0.008 for all comparisons). The maximal number of migrated DCs was 11,496 for the F3, and 9,152 for the NH36 vaccine (Figure 2C). Accordingly, 75% of the migrated DCs count of the F3 vaccine group fall below 8,596, while 75% of the NH36 vaccine counts fall only below 6,130 DCs counts. Therefore, although the differences in the generation of the DC migrating capabilities of the two vaccines were not statistically significant, the maximal values and 75th percentiles suggest the superiority of the F3 vaccine. As a blocking control, we incubated DCs obtained from either infected or F3sap-vaccinated and challenged mice, with anti-CCR7 antibody, in the presence of CCL19 stimulus (Figure 2D). The anti-CCR7 antibody blocked 90% of the *in vitro* migrating capabilities of the DCs obtained from the F3sap-vaccinated mice.

Our results indicate that protective immunity against the F3 and NH36 antigens, not only restores the DCs migrating dysfunction of mice infected with *L. (L.) infantum chagasi*, but it also potentiates this capability above the levels found in normal naïve mice (Figure 2). In agreement with the parasitological results, which indicated that the F3 vaccine was the most protective, the superiority of the F3 vaccine is reinforced by the finding of a higher number of purified DCs, higher maximal counts of migrated DCs, and strong inhibition of these migrating capabilities by treatment with anti-CCR7 antibody.

**Figure 3:**
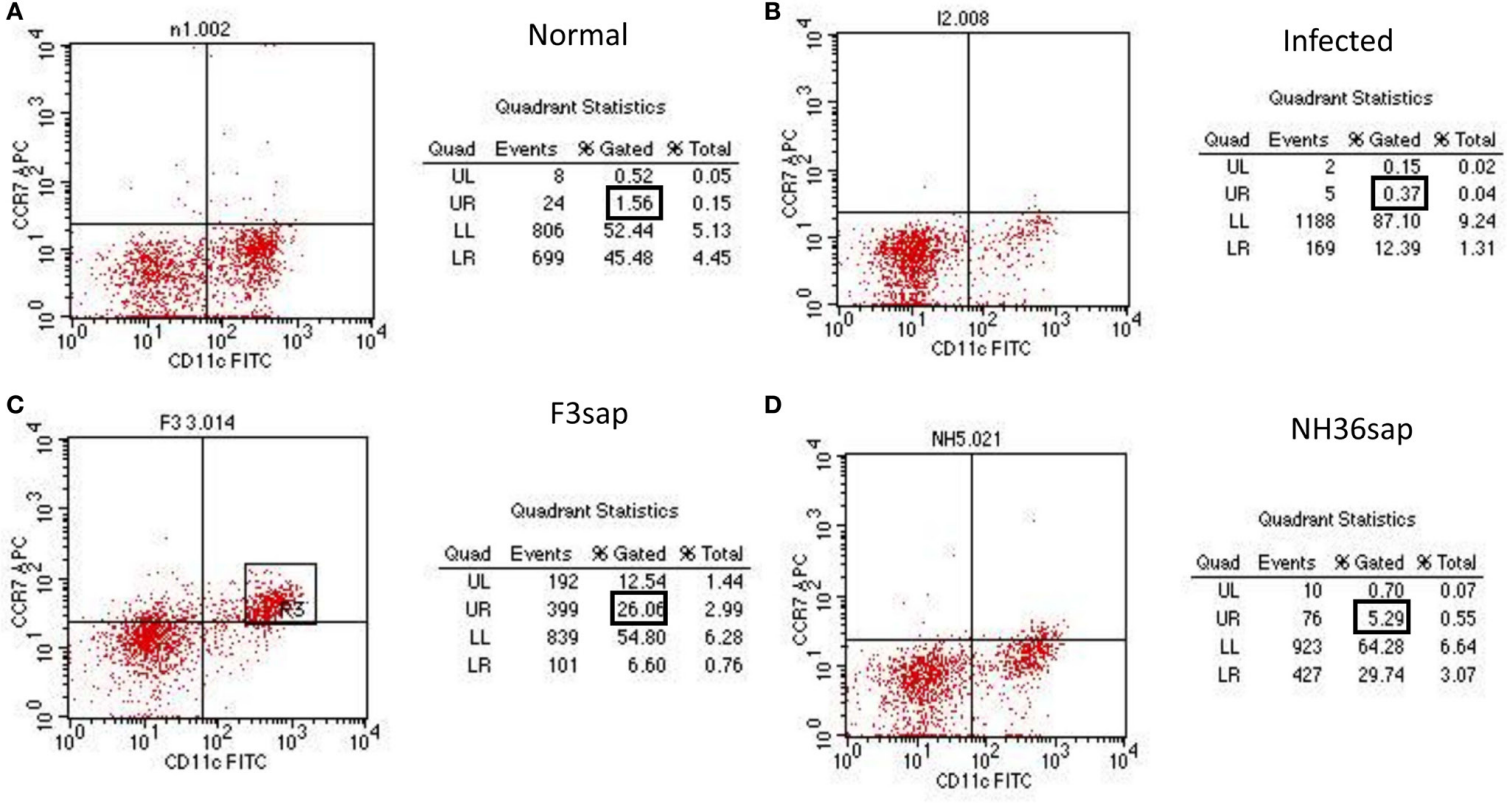
Enhanced CCR7^+^ expression in F3 and NH36-primed dendritic cells (DCs). DCs from normal **(A)**, infected **(B)**, and F3 **(C)** or NH36 **(D)** vaccinated and challenged C57BL/6 mice were purified and analyzed by flow cytometry. DCs CD11c^+^ populations were gated and analyzed for CCR7 expression using anti-CCR7-allophycocyanin antibody. Results are representative of the individual percentages gated of two experiments (*n* = 5 per treatment for each experiment).

In VL, abnormal DC migration is partially due to the reduced expression of their CCR7 receptor, which is sensitive to the CCL19 chemokine. In order to evaluate whether the restored DCs migrating capabilities of vaccinated mice were related to a preserved CCR7 expression on DCs. With that aim, we analyzed the CCR7 protein expression on the surface of purified splenic DCs using APC-conjugated anti-CCR7 antibody and flow cytometry analysis. Based on our findings (Figure 3), vaccination with F3sap and NH36 upregulated the expression of CCR7 receptor on DCs. CCR7 expression was low in normal mice (1.56%) (Figure 3A), extremely reduced in infected mice (0.37%) (Figure 3B), and much stronger in animals treated with the F3 vaccine (26.06%) (Figure 3C) than in those vaccinated with NH36 (5.29%) (Figure 3D).

Therefore, vaccination with F3 and NH36 stimulates IDR, protects mice from clinical VL, restores and enhances DCs migrating capabilities probably due to the increase in CCR7 expression on DCs. Our findings of the reduced liver parasite load, increased IDR and enhancement of the CCR7 expression highlight the stronger immunogenic effect of the F3 vaccine.

### Immunotherapy With DCs

**Figure 4:**
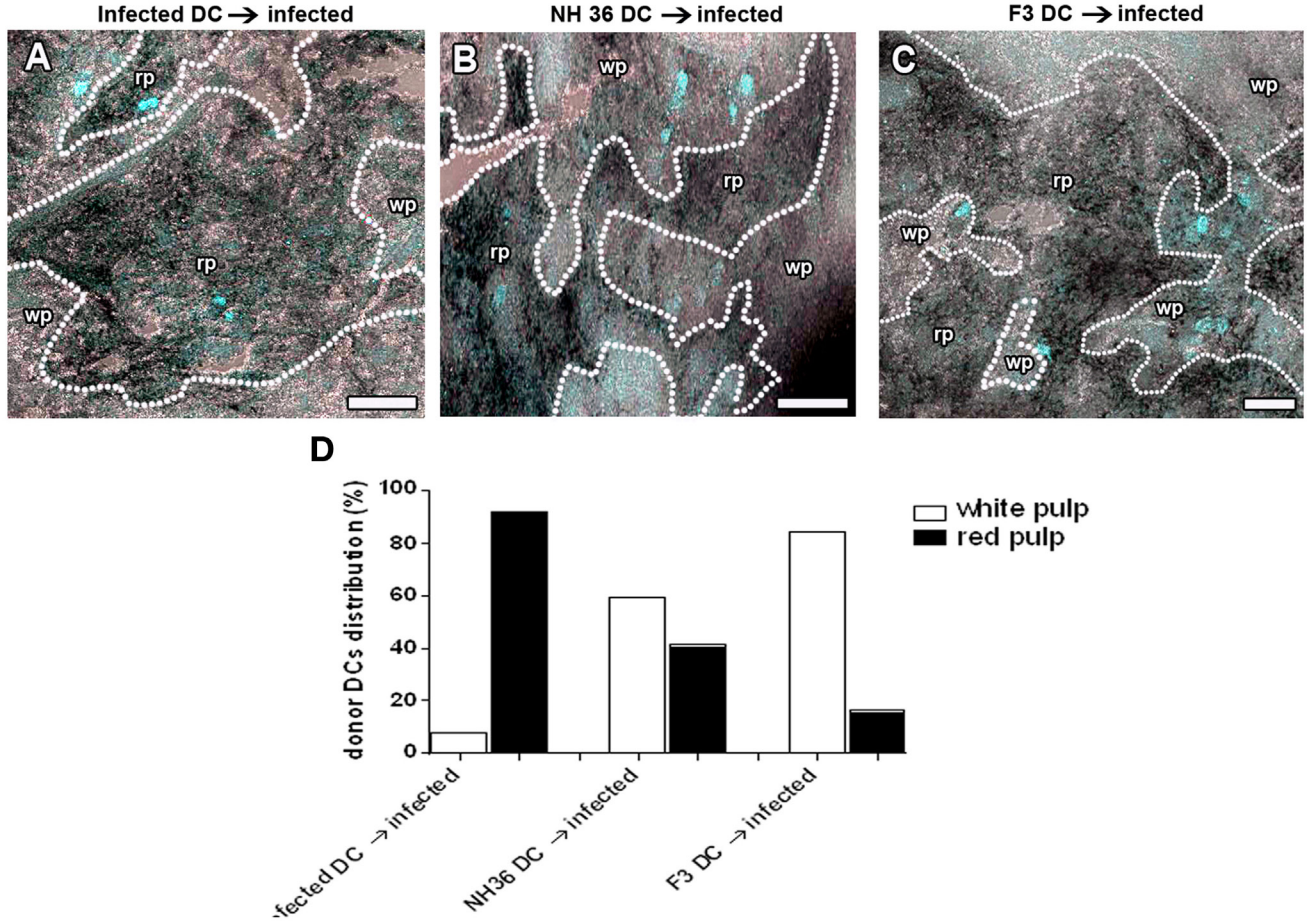
F3-primed dendritic cells (DCs) migrate to the spleen white pulp (wp) of infected mice. DCs of day 28-infected mice and F3- and NH36-primed and -challenged mice were labeled with Hoescht nuclear fluorescent stain, and injected into 28-day-infected mice. Recipient mice were euthanized 24 h after injection. Spleens were fixed and frozen and the distribution of stained DCs was assessed in cryostat sections to confirm the *in vivo* migration of DCs. We show the image overlay of cryostat sections from bright field to evidence spleen morphology, and confocal image to show Hoescht labeled cells in blue. The dotted lines indicate the marginal zone (MZ) which separates the red pulp (rp) from the wp. It is possible to observe the localization of the labeled DCs in mice that received DCs from infected **(A)**, NH 36- **(B)**, or F3-vaccinated donors **(C)** as well as the distribution of these cells **(D)**. Scale bar = 120 µm. Data are representative of one experiment with three mice per treatment. DCs from infected donors remain mostly in the rp (92%; *p* < 0.001). DCs from NH36-vaccinated mice are equally distributed between the white and the rp (*p* = 0.1144) while 84% (*p* < 0.0001) of DCs from F3-vaccinated mice migrated to the wp and its MZ **(C)**.

In order to confirm the DC migration *in vivo*, DCs from infected, or NH36- or F3-vaccinated and *L. (L.) infantum chagasi*-challenged mice, were stained with Hoescht 33342 *in vitro*, and further injected into infected mice. Twenty four hours after injection, the spleens of receptors were removed and frozen, and the transferred DCs were observed by fluorescence microscopy in the wp or rp of the frozen sections (Figure 4). Fluorescent DCs were counted in a total of 19–21 sections of each treatment and representative images are shown in Figures 4A–C. Additionally, the records are represented as proportions of DCs from donors distributed either in the white or rps of infected receptor spleens. On day 28 of the infection of recipients, DCs from infected donors remained mostly in the rp (92%) (Figure 4A) and, only minor proportions migrated to the MZ of the wp (8%; *p* < 0.001). DCs from NH36-vaccinated mice are equally distributed between the white (59%) and the rp (41%; *p* = 0.1144) (Figure 4B). Remarkably, 84% of DCs from F3-vaccinated mice preferentially migrated to the wp and its MZ (Figure 4C), while only 16% remained in the rp (*p* < 0.0001).

**Figure 5:**
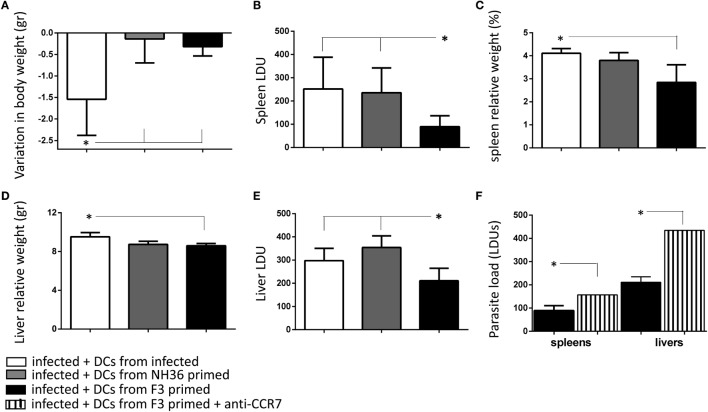
Immunotherapy with F3-primed dendritic cells (DCs) reduces parasite and clinical signs of visceral leishmaniasis. DCs (10^6^) purified from *Leishmania (L.) infantum chagasi*-infected mice, or mice treated with the F3 or the NH36 vaccines and challenged, were injected into infected mice on day 28 after infection. Recipient mice were euthanized after 7 days after DC transfer and the variation in corporal weight **(A)**, spleen LDU **(B)**, spleen/body relative weight **(C)**, liver LDU **(D)**, and liver/body relative weight **(E)** were calculated. As a control we also showed the increase of the parasite load of spleens and livers of day 28-infected mice that received an injection of F3-primed DCs pre-incubated with anti-CCR7 antibody **(F)**. The results are compared with the parasite load of infected mice that received F3-primed DCs with no previous incubation. Bars represent the mean + SE values of two-independent experiments (*n* = 5 mice per treatment in each experiment). Asterisks and horizontal lines show significant differences between treatments as disclosed by Mann–Whitney non-parametrical test.

In order to study the potential use of F3 and NH36 antigen-primed DCs in the immunotherapy of VL we transferred DCs from infected, and from vaccinated and challenged mice, into recipient mice infected with *L. (L.) infantum chagasi* 28 days before. Seven days after the DCs transfer, the impact on the parasitological and clinical cure was assessed. While infected animals lost 1.54 g of corporal weight, the weight loss was 79% lower in mice that received F3-primed DCs (0.32 g), and 91% lower in mice treated with NH36-primed DCs (0.14 g) (Figure 5A). Additionally, only the F3-primed DCs were able to alter the impact of the infection on mice, by reducing the parasite load in the spleen (*p* < 0.007) (Figure 5B), the spleen/body relative weight (Figure 5C), the liver/body relative weight (*p* < 0.05) (Figure 5D), and the liver LDU values (*p* < 0.05) (Figure 5E). Therefore, the results of immunotherapy with DCs correlate with the results of the preventive vaccination, thus demonstrating that the F3 vaccine is more potent than the NH36 vaccine. The immunotherapeutic effect of F3-primed DCs (Figure 5F), but not of the NH36-primed DCs (not shown) was blocked by pre-incubation with anti-CCR7 antibody, and a 43 and 52% increased parasite load was observed in spleens and livers, respectively of the recipient mice. These results indicate that the increased migrating capabilities of DCs from F3-vaccinated mice, both *in vitro* and *in vivo* (Figures 2D, 4C and 5F) are related to the enhanced CCR7 receptor expression (Figure 3), which contributes to the cure of VL.

### Evolution of the Cellular Immune Response of Vaccinated DC Donor Mice Secreted Cytokines and Survival

**Figure 6:**
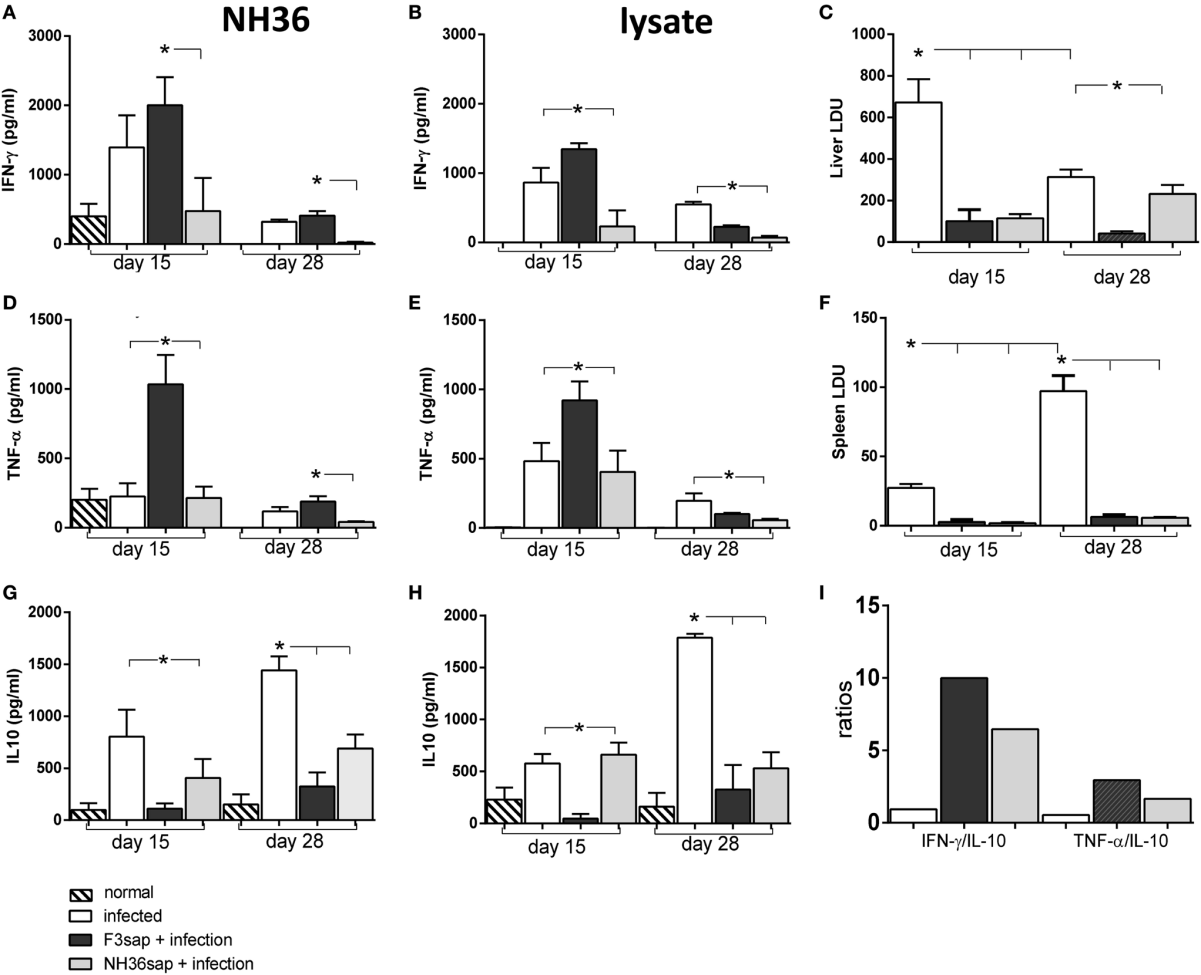
Cytokines secreted by splenocytes along the infection. Splenocyte secretion of IFN-γ **(A,B)**, TNF-α **(D,E)**, and IL-10 **(G,H)** were measured in supernatants of *in vitro* cultures of infected, F3sap and NH36sap vaccinated and infected, in response to NH36 or *Leishmania (L.) infantum chagasi* lysate antigens, both on days 15 and 28 after infection. LDU values show that the parasite load is maximal in livers at day 15 **(C)** and in spleens, only on day 28 **(F)**. Bars represent the mean + SE values of two-independent experiments (*n* = 5 mice per treatment in each experiment). Asterisks and horizontal lines show significant differences between treatments as disclosed by Mann–Whitney non-parametrical test. IFN-γ/IL-10 and TNF-α/IL-10 ratios in response to NH36 were calculated on day 15 **(I)**.

We assessed the profile of IFN-γ, TNF-α, and IL-10 secretion along the time, in the supernatants of splenocytes of mice vaccinated with F3 or NH36 vaccines and further challenged (Figure 6). We monitored the cytokine secretion of splenocytes in response to NH36 or to the *L. (L.) infantum chagasi* lysate antigen. On day 15 after challenge, when the parasite load was maximal in livers (Figure 6C), the F3-vaccine enhanced the IFN-γ and TNF-α secretion in response to NH36, five times more than the NH36 vaccine (Figures 6A,D). The F3-vaccine also enhanced by a factor of 1.4–5, the respective IFN-γ and TNF-α levels when compared to infected controls (Figures 6A,D). On the other hand, IL-10 secretion was reduced more by the F3 than by the NH36 vaccine (Figure 6G). The IFN-γ/IL-10 and TNF-α/IL-10 ratios (Figure 6I), that were also higher for the F3 vaccine (10 and 2.9, respectively) than for the NH36 vaccine (6.5 and 1.7, respectively) suggest the superiority of the F3sap vaccine in the induction of a probable Th1 response. The *Leishmania* lysate promoted a similar response with superiority of the F3 vaccine although with a lower IFN-γ on day 15 and higher IL-10 secretion on day 28 (Figures 6B,G). Globally, the pro-inflammatory cytokine response of splenocytes was maximal (Figures 6A,B,D,E) since the early infection, when vaccine protection was already registered in spleens (Figure 6F). Levels of IFN-γ and TNF-α decreased until day 28, when, in contrast, IL-10 secretion was maximal. This pike in IL-10 secretion is coincident with the maximal parasite load detected in spleens. However, the vaccine efficacy was also long-lasting until day 28, when the parasite load was higher in spleens. In fact, while 90% of the parasite load reduction was induced by the F3 vaccine on day 15, protection was maximal (94%) on day 28 after infection (Figure 6F).

**Figure 7:**
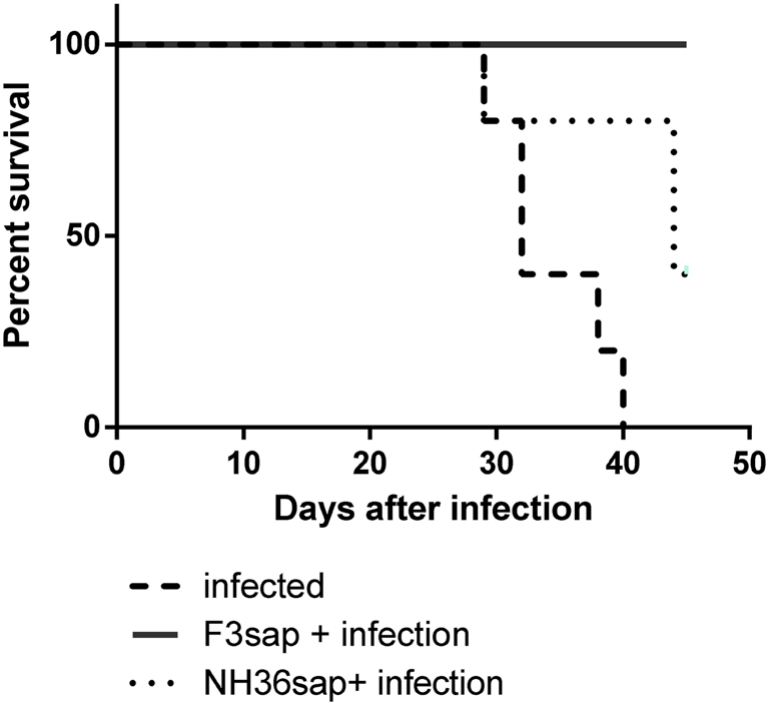
Longevity of vaccine efficacies. The survivability of infected, F3sap, and NH36sap vaccinated and infected mice were recorded and represented using a Kaplan–Meir curve. Comparison of the survival distribution of infected controls and vaccinated and challenged mice, using the Log-rank Mantel–Cox and the Gehan–Breslow–Wilcoxon tests disclosed significant differences for the F3sap vaccine (*p* = 0.0018 and *p* = 0.0039, respectively). Differences between controls and the NH36sap vaccine were, however, only detected by the Log-rank Mantel–Cox (*p* = 0.0277).

The survival Kaplan–Meier curve analysis represented in Figure 7, confirmed that protection generated by the F3sap vaccine is long-lasting. In fact, while all mice from the infected control group died between day 29 and day 40 after infection, a 100% of mice vaccinated with F3sap survived until euthanasia on day 45, confirming the longevity of the vaccine immune response. In contrast, 80% of NH36-vaccinated mice survived until day 29 and only 40% of them were alive on day 45 (Figure 7).

**Figure 8:**
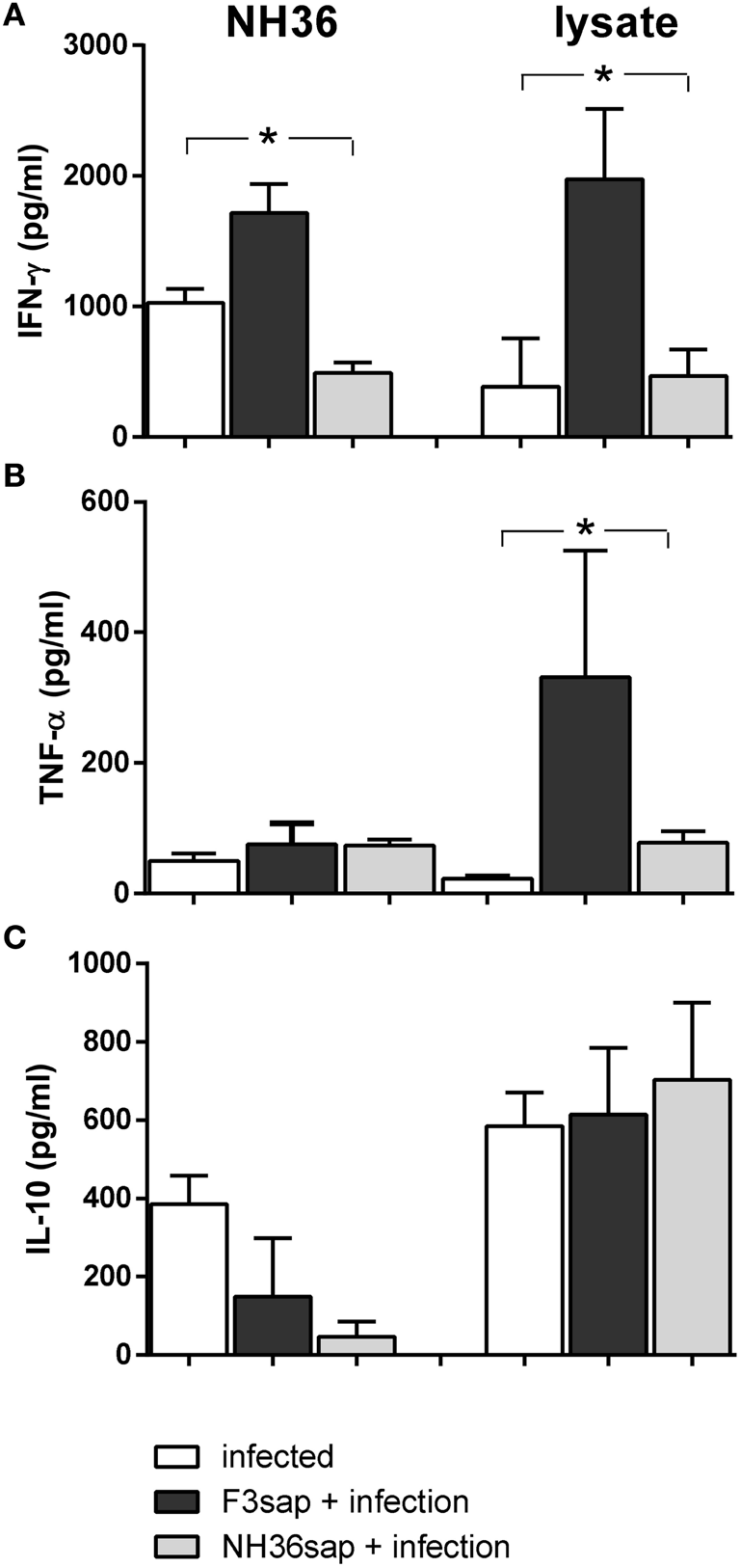
Cytokines secreted by dendritic cells (DCs) on day 28 after challenge. Secretion of IFN-γ **(A)**, TNF-α **(B)**, and IL-10 **(C)** were measured in supernatants of *in vitro* cultures of purified DCs obtained from infected, F3sap, and NH36sap vaccinated and infected in response to NH36 or *Leishmania (L.) infantum chagasi* lysate antigens, on day 28 after infection. Bars represent the mean + SE values of two-independent experiments (*n* = 5 mice per treatment in each experiment). Asterisks and horizontal lines show significant differences between treatments as disclosed by Mann–Whitney non-parametrical test.

We also studied the cytokine response *in vitro*, in the supernatants of DCs obtained from infected, and vaccinated and infected DC donors, on day 28, before transfer to infected mice (Figure 8). Although the total splenocyte cytokine response was lower on day 28, than on day 15 (Figures 6A,B,D,E,G,H), the DCs of F3-vaccinated mice secreted enhanced levels of IFN-γ, in response to NH36 and lysate (Figure 8A) and of TNF-α, in response to lysate only (Figure 8B). No significant differences in IL-10 levels were observed in response to the antigens (Figure 8C).

### Intracellular Expression of Cytokines

The Figure S2 in Supplementary Material, summarized the strategy used for the analysis of multifunctional T cell response using a four-color flow cytometry panel to simultaneously analyze multiple cytokines at the single-cell level in splenocytes cultures. After analyzing the total production of each cytokine by CD4^+^ or CD8^+^ cells, the Boolean gates (or combinatorial analysis) tool of the FlowJo program was used to determine the frequencies of the possible seven combinations of cytokine-producing cells.

**Figure 9:**
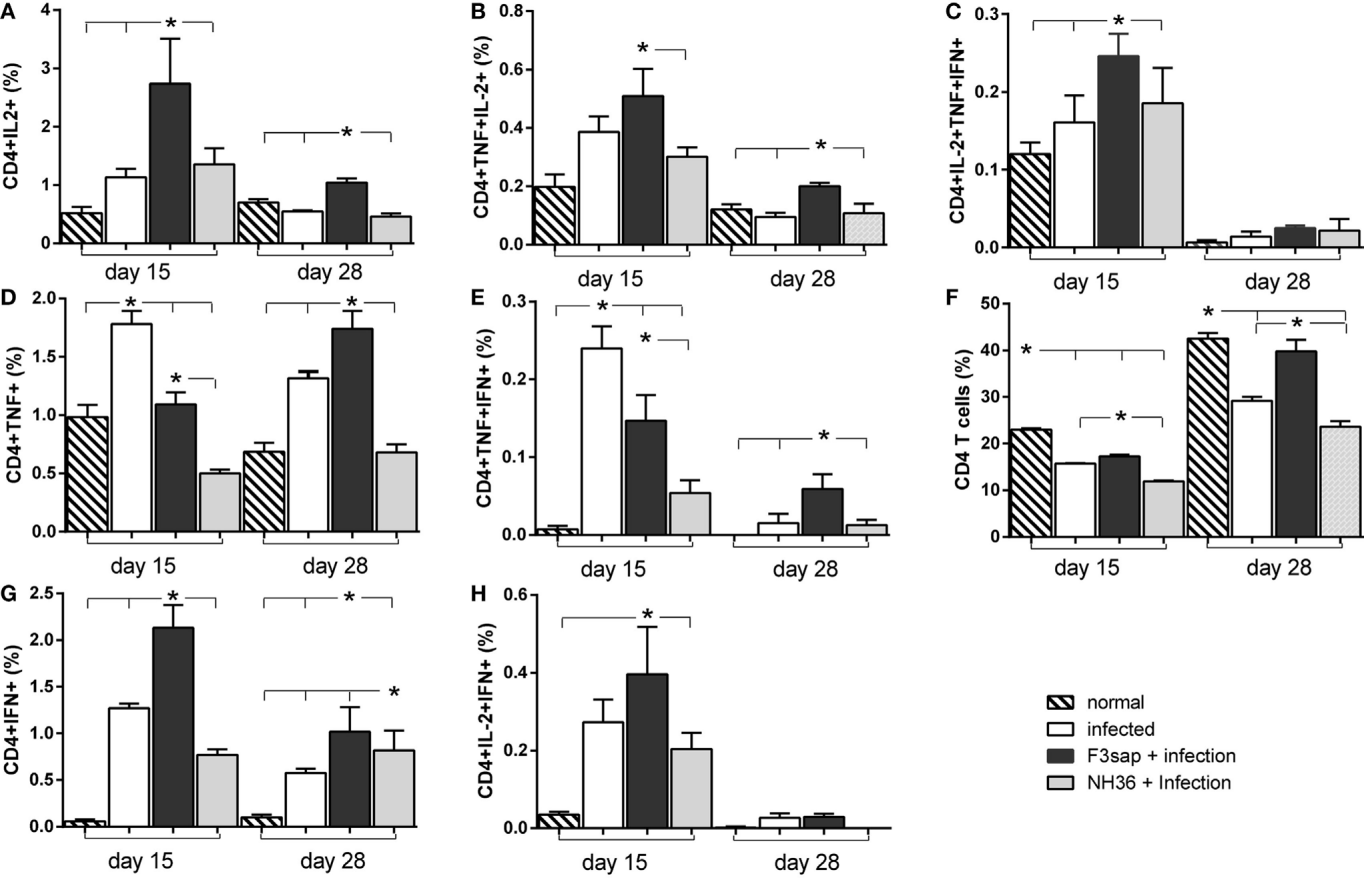
Cytokines expressed by CD4^+^ T lymphocytes in response to NH36. Effect of the F3 and NH36-vaccines on the frequencies of CD4^+^IL-2^+^ **(A)**, TNF-α^+^ **(D)**, IFN-γ^+^ **(G)**, TNF-α^+^IL-2^+^ **(B)**, TNF-α^+^IFN-γ^+^ **(E)**, IL-2^+^IFN-γ^+^ **(H)**, and IL-2^+^TNF-α^+^IFN-γ^+^-secreting T cells **(C)** in response to the NH36 antigen, on day 15 and 28 post challenge. The total CD4^+^ T cell frequencies are also represented **(F)**. Bars represent the mean + SE values of two-independent experiments (*n* = 5 mice per treatment in each experiment). Asterisks and horizontal lines show significant differences between treatments as disclosed by Mann–Whitney non-parametrical test.

In correlation with the cytokine secretion to supernatants by splenocytes (Figure 6), the intracellular expression of cytokines by CD4^+^ T lymphocytes in response to NH36, was more intense on day 15 than on day 28 after infection (Figure 9). On both days, the F3sap vaccine was superior to the NH36 formulation enhancing all types of CD4^+^ T cells secreting one cytokine (IL-2^+^, TNF-α^+^, or IFN-γ^+^) (Figures 9A,D,G), the combination of two (IL-2^+^TNF-α^+^, TNF-α^+^IFN-γ^+^, or IL-2^+^IFN-γ^+^) (Figures 9B,E,H), or the three cytokines simultaneously (IL-2^+^TNF-α^+^IFN-γ^+^) (Figure 9C). The only exception was detected in CD4^+^TNF-α^+^ (Figure 9D) and CD4^+^TNF-α^+^IFN-γ^+^ T cells (Figure 9H) which were increased more, in the infected controls than in the F3-vaccinated mice, only on day 15.

Additionally, the global proportions of CD4^+^ T lymphocytes were lower in infected, than in normal mice (Figure 9F), although the F3 vaccine sustained higher CD4^+^ T cells counts than the NH36 vaccine, even despite the advancement of spleen infection on day 28 (Figure 6F).

In contrast to the IFN-γ and TNF-α splenocyte secretion to supernatants (Figure 6), which were slightly higher in response to NH36 than to the lysate (Figure 6), the intracellular expression of cytokines in CD4^+^ T cells was similar, in response to both antigens (Figure 9; Figure S3 in Supplementary Material). In fact, the *Leishmania* lysate stimulus also increased the frequencies of CD4^+^-secreting T cells, more on day 15 than on day 28 after infection. The F3 vaccine was stronger than the NH36 vaccine for all types of CD4^+^ T cells (Figure S3 in Supplementary Material). Also, and as detected after stimulation with NH36, the frequencies of CD4^+^TNF-α^+^ and CD4^+^TNF-α^+^IFN-α T cells were still higher in infected controls and only increased by the F3-vaccine, on day 28 after infection (Figures S3D,E in Supplementary Material).

**Figure 10:**
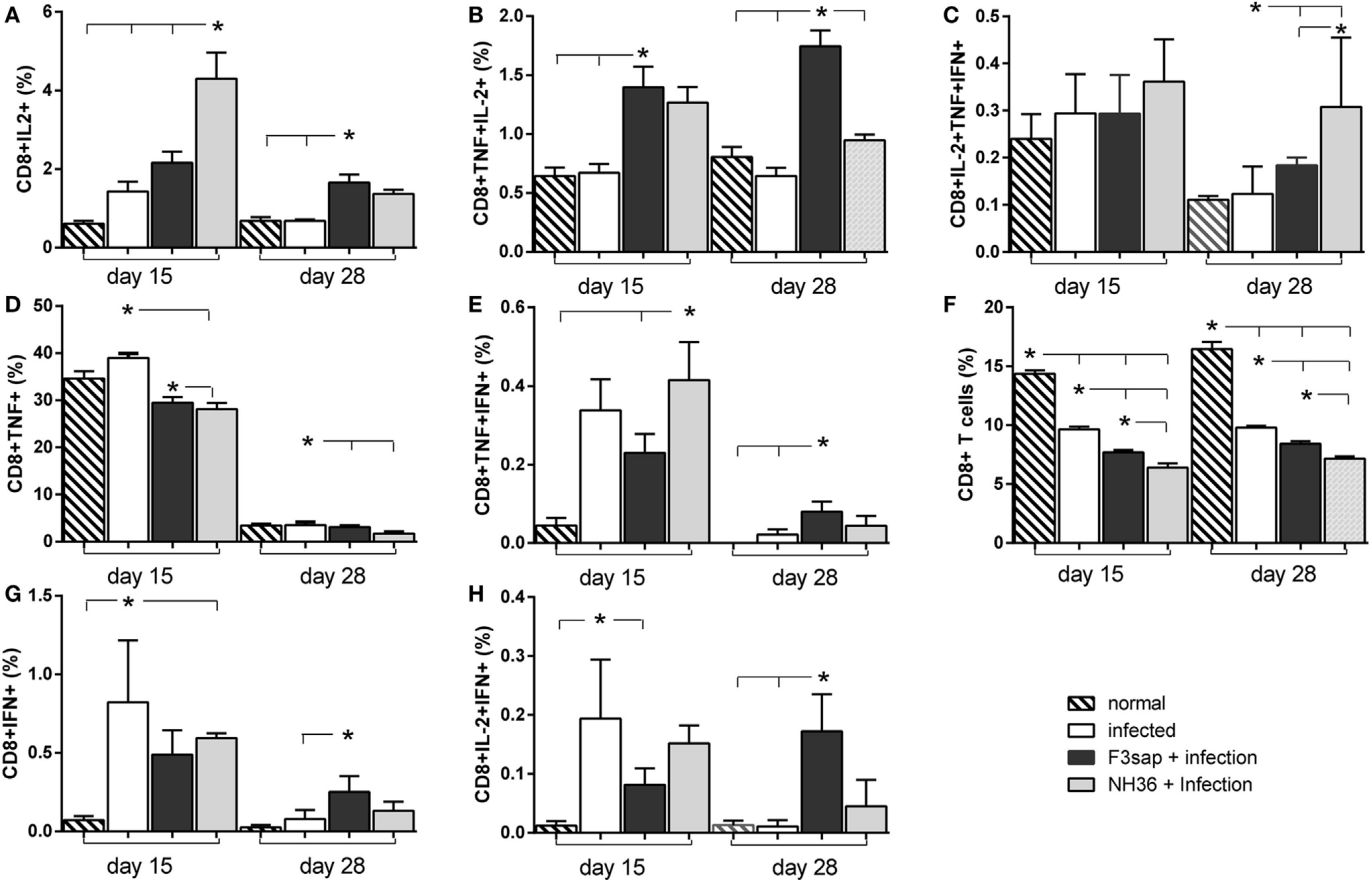
Cytokines expressed by CD8^+^ T lymphocytes in response to NH36. Effect of the F3 and NH36-vaccines on the frequencies of CD8^+^IL-2^+^ **(A)**, TNF-α^+^ **(D)**, IFN-γ^+^ **(G)**, TNF-α^+^IL-2^+^ **(B)**, TNF-α^+^IFN-γ^+^ **(E)**, IL-2^+^IFN-γ^+^ **(H)**, and IL-2^+^TNF-α^+^IFN-γ^+^-secreting T cells **(C)** in response to the NH36 antigen, on day 15 and 28 post challenge. The total CD8^+^ T cell frequencies are also represented **(F)**. Bars represent the mean + SE values of two-independent experiments (*n* = 5 mice per treatment in each experiment). Asterisks and horizontal lines show significant differences between treatments as disclosed by Mann–Whitney non-parametrical test.

Regarding the cytotoxic immunity in response to NH36, the NH36-vaccine was the strongest enhancer of the proportions of CD8^+^IL-2^+^ and CD8^+^TNF-α^+^IFN-α^+^ on day 15 (Figures 10A,E) and of CD8^+^IL-2^+^TNF-α^+^IFN-γ^+^ lymphocytes on day 28 (Figure 10C). In contrast, on day 28, the F3-vaccine was the most potent enhancer of the frequencies of CD8^+^IL-2^+^ (Figure 10A), IFN-γ^+^ (Figure 10G), TNF-α^+^IL-2^+^ (Figure 10B), TNF-α^+^IFN-γ^+^ (Figure 10E), and IL-2^+^IFN-γ^+^-secreting T cells (Figure 10H). The F3-vaccine superiority for TNF-α^+^IL-2^+^ enhancement was already evident on day 15 (Figure 10B).

Additionally, when stimulated with lysate stronger frequencies of CD8^+^IL-2^+^, CD8^+^TNF-α^+^IL-2^+^, CD8^+^TNF-α^+^IFN-γ^+^, and CD8^+^IL-2^+^TNF-α^+^IFN-γ^+^ T cells were observed, on day 15 (Figure S4 in Supplementary Material) in mice vaccinated with the NH36 vaccine. In contrast, the F3-vaccine was superior for the proportions of CD8^+^IFN-γ^+^ and CD8^+^IL-2^+^IFN-γ^+^ T cells, both on days 15 and 28, and of CD8^+^IL-2^+^ and CD8^+^IL-2^+^IFN-γ^+^ T cells, on day 28. Similar to what was detected for CD4^+^ T cell whole frequencies (Figure 9F) the CD8^+^ T cell proportions were reduced in all challenged mice, but in this case, no preventive effective was induced by any of the vaccines (Figure S4F in Supplementary Material).

## Discussion

Although no human vaccine is available for human VL yet, there are four veterinary vaccines that have been developed for prophylaxis of canine VL (30–33). FML, a complex glycoprotein antigen of *L. (L.) donovani* and saponin, are the components of Leishmune^®^, the first licensed canine vaccine against leishmaniasis (33–35). The Leishmune^®^ dog vaccination has decreased the incidence of canine and human leishmaniasis in endemic areas (33). Leishmune^®^ is a transmission blocking vaccine (27, 34) and vaccinated dogs remained non-infective to insect vectors (35). NH36 is the main antigen of FML (27, 36) and F3 is its C-terminal domain (15). The F3-vaccine induced a significantly higher decrease in parasite load (95%) and splenomegaly (49%) in C57Bl6 mice infected with *L. (L.) infantum chagasi* (14) than the NH36 vaccine, which in contrast, reduced the parasite load and relative weight by 87 and 39%, respectively (14). Both vaccines, however, prevented, to a similar extent, the increase in total counts of DCs and severe splenomegaly due to *L. (L.) infantum chagasi* infection (14), both of which were also previously observed during infections with *L. (L) donovani* in BALB/c and C57BL/6 mice (11).

In this investigation, we demonstrate that both NH36 and F3 vaccines increased the IDR, strongly reduced the parasite load in livers and the liver/body relative weight and promoted a gain in corporal weight. We also showed that IDR was also a strong correlate of protection against VL (15). Noteworthy, F3 was more potent than the NH36-vaccine in IDR after *L. (L.) infantum chagasi* challenge and also in reducing the liver parasite load of C57Bl6 mice. The importance of NH36 in immune prevention against VL has been extensively proven in mice (15, 16, 18, 21), dogs (17), and suggested for humans (19, 20, 37). Nonetheless, we also demonstrated that the F3 domain is the responsible for the CD4^+^ T cell-mediated protection induced by NH36 (15). In fact, F3 was 37% more powerful than the NH36 cognate protein in the increase of antibodies, frequencies of CD4^+^ T lymphocytes, levels of secreted IFN-γ, and ratios of CD4^+^ and CD8^+^ T lymphocytes producing IFN-γ and IL-10 (15) and holds the most potent antibody (15, 38) and MHC class II restricted epitopes (15, 21). In that investigation (15), IDR and ratios of TNFα/IL-10 producing CD4^+^ T cells were strong correlates of protection. Further, *in vivo* depletion with anti-CD4 and anti-CD8 monoclonal antibodies confirmed protection, which was also, long-lasting. The F3 domain was also responsible for the CD4^+^ T cell-mediated protection against mice infection by *L. (L.) amazonensis* (21).

In this investigation, our results confirm the superior efficacy of the F3-vaccine in inducing a Th1 response against *L. (L.) infantum chagasi* infection in C57Bl6 mice, which includes the generation of long-lasting protection. The F3 vaccine induced an earlier protective T cell response (day 15) with multifunctional CD4^+^IL-2^+^TNF-α^+^IFN-γ^+^ T cells, while the NH36 vaccine promoted a CD8^+^IL-2^+^TNF-α^+^IFN-γ^+^ T cell response, which started later (day 28). Besides the multifunctional T cells, the F3 vaccine also increased the proportions of CD4^+^ T cell secreting IL-2 or IL-2 and TNF-α, and the NH36 vaccine enhanced the frequencies of CD8^+^ T cell secreting IL-2 or IL-2 and TNF-α. These cells have been considered as a reservoir of memory T cells that have also effector potential and that can, together with the multifunctional T cells, promote optimal protection (39).

Additionally, the response of CD4^+^-secreting T cells was slightly higher for the NH36 than to the lysate stimulus, indicating that the NH36 is an important and predominant antigen of *Leishmania* promastigotes. The cytotoxic response, in contrast, is more stimulated by the lysate than by the NH36 antigen, and stronger in mice vaccinated with the NH36, than with the F3 vaccine. These results suggest that the CD8 response is directed to epitopes of NH36, which are not located in the F3 domain. As a matter of fact, the most important epitope for MHC class I molecules of NH36 is the YPPEFKTKL epitope of the F1 domain, which was also shown to be responsible for IL-10 secretion (21). In this way, this F1 domain-epitope contributes to the lowest IFN-γ and TNF-α/IL-10 ratios found in supernatants of splenocytes of NH36-vaccinated mice.

Some of the evidence gathered in this investigation pointed out the superiority of the F3 vaccine, which correlated with the parasitological results. Among them, we can consider the findings of: (1) higher numbers of purified DCs in F3-vaccinated mice; (2) higher maximal counts of migrated DCs; (3) stronger inhibition of these migrating capabilities *in vitro* and *in vivo* by treatment with anti-CCR7 antibody, and (4) higher expression of CCR7, detected on DCs of vaccinated mice. This shows why, the F3 vaccine protects mice from the defective migration of DCs. The higher expression of CCR7 receptors on DCs and the stronger migration of DCs to the splenic wp, induced by the F3-vaccine, explain the triggering of the most potent CD4-Th1 immune response. This immune response determines the cure of VL, as evidenced by the significant reduction of spleen and liver parasite load and spleen and liver/relative weights, the long-lasting immunity and 100% of survival. On the other hand, NH36-primed DCs, which showed lower expression of CCR7 receptors and consequently lower migration capabilities (11), did not show an effective cure of VL. However, the direct effect of protection offered by F3 vaccination *via* DC-mediated mechanism against other *Leishmania* species, agents of visceral or cutaneous leishmaniasis, has not yet been investigated and this is a limitation.

The CCR7 expression on the surfaces of DCs is mandatory to ensure their correct movement toward lymph nodes. The geographical encounter of DCs and naive T lymphocytes occurs in secondary lymphoid organs (40). This important event is compromised in VL. The main mechanism that promotes the defective localization of DCs is dependent on the IL-10-mediated inhibition of the CCR7 expression. IL-10 reduced migration of DCs by 47% and decreased the DCs CCR7 expression, in *L. (L.) donovani*-infected mice (11). Treatment with anti-IL-10 monoclonal antibody even restored DC migration (11). We recently showed in BALB/c mice that, in contrast to the high IL-10 secretion observed in infected animals, F3-vaccinated and challenged mice showed absolutely no secretion of IL-10 (15, 21). In fact, we described that the FRYPRPKHCHTQVA and KFWCLVIDALKRIG CD4-epitopes of F3, promoted the secretion of TNF-α, but not of IL-10 (21). In the present investigation, secretion of IL-10 was present in infected animals, but was more reduced by the F3 than by the NH36 vaccine, as observed by their respective IFN-γ/IL-10 and TNF-α/IL-10 ratios, which characterize a Th1 response against the infection. These results can partially explain the sustained high CCR7 expression on DCs of thr F3-vaccinated mice and their correct migration capabilities.

In VL, the protective response to VL is compromised by the functional impediment of DCs, which no longer move toward the areas of secondary lymphoid organs, where T cells are located (11). This defect restricts the initial specific-T cell response to *Leishmania* infection (41). Immunization with NH36 and F3 were able to restore the functional impairment of CD11c^+^ DCs by inducing CCR7/CCL19-mediated responses and protective immunity in VL. We explored the possible use of mature DCs obtained from mice previously immunized with the C-terminal F3 or the NH36 antigens, to recover protective responses in *L. (L.) infantum chagasi-*infected mice. Our results indicate a clear immunotherapeutic effect in mice receiving the F3 antigen-primed DCs.

The work of Ato et al. (11), showed that normal naïve DCs preserve their migration capabilities and, in this way, help in immunotherapy of VL. However, they used DCs pre-pulsed with LPS and/or antigen to obtain a reduction of the spleen parasite load (11). We now showed that DCs of F3-vaccinated mice enhanced by 58% the migrating capabilities of DCs from normal mice *in vitro*, and without antigen or LPS pre-incubation, and also determined a 65% of reduction of spleen parasite load *in vivo*. While only 70% of DCs of normal naïve mice migrate to the wp of *L (L.) donovani*-infected mice (11), we demonstrated that 84% of DCs from F3-vaccinated mice preferentially migrated to the wp of *L. (L.) infantum chagasi*-infected mice. These effects explain the F3-vaccine usefulness and its higher efficacy in immunotherapy of VL, long-lasting preventive immune response and the 100% survival.

NH36 is a recombinant protein that was first described as the main native glycoprotein GP36 of the FML extract (42). The GP36-glycidic moiety is composed of short chains of 4-O-mannopyranose alternating with 3-O and 4-O-substituted fucopyranose residues (27) and its antigenicity is abolished by treatment with sodium m-periodate (27, 42). We, and others, further identified and cloned the NH36 gene and described its peptide sequence (36, 43). NH36 recombinant protein is a very strong and specific protein diagnostic antigen for human and canine VL (36, 44) and is recognized by mice antibodies (15, 21, 26). We proved that vaccination with the NH36 recombinant protein or DNA protect mice from visceral (15, 16, 18) and cutaneous leishmaniasis (16, 21, 26, 45), and controls canine VL (17). NH36 protein sequence, obtained in *E. coli* is now considered worldwide, as a very potent *Leishmania* antigen and a good candidate for an animal and human vaccine against leishmaniasis (15–21, 26, 37, 38, 45). Recently, the NH36 sequence was analyzed and four N-glycosylation sites represented by asparagine (Q) were found (46), but only two of them of high predictive value. As expected, the further cloning in *Pichia pastoris* enhanced the expressed protein yield but also, apparent molecular weight of NH36, suggesting the presence of high mannose chains (46). However, in order to avoid the glycoside moiety interference to the peptide antigenicity, the asparagine residues were mutated, and still, the un-glycosylated NH36 remained strongly antigenic (46).

Although the GP36 antigen in its native form, exhibits a glycidic moiety (27), it is the NH36 protein that is explored as an extremely successful antigen, and is the tool developed for effective vaccination (15–21, 26, 37, 38, 45). We showed that F3 is the strongest NH36 domain to be used in vaccination and, supporting our strategy, none of the four potential sites of glycosylation of NH36 are located in the F3 domain (amino acids N39, N77, N89, and N189) (46). Therefore, there would be no influence of any carbohydrate native moiety in the native form of the F3 antigen.

Trying to assess which are the receptors for F3 on DCs we previously studied the potential contribution of G-protein-coupled kinin receptors (B2R) in the protective immunity against mice VL induced by the F3-saponin vaccine (47). B2R^−/−^ and wild type C57BL/6 controls (B2R^+/+^) were vaccinated with F3 and saponin, challenged with amastigotes of *Leishmania (L.) infantum chagasi* and euthanized 30 days later. The IDR to leishmanial from B2R^−/−^ vaccinated mice was lower than in wild type controls. Additionally, a significant decrease of 44.7% of spleen relative weight was noted in vaccinated B2R^+/+^, but not in vaccinated B2R^−/−^ mice, indicating that the B2R of kinin, present on the surface of DCs cells has a partial contribution to the protection against splenomegaly, induced by the F3 vaccine (47). The G protein-coupled bradykinin type 2 receptor (B2R) may couple different classes of G proteins and simultaneously initiate different signal chains, which have extensive cross-talk. In addition, the B2R receptor can generate mitogenic signals that involve the mitogen-activated protein kinases and transactivation of receptor tyrosine kinases (48). Therefore, the signaling pathway activated after recognition of F3 deserves further studies.

Additionally, MHC class II receptors on the surface of DCs of mice are potential targets of recognition of the epitopes of NH36. We recently described the induction of a mixed Th1/Th2 immunity in response to the FMLQILDFYTKVYE of F3, and in contrast, the generation of a main Th1 response, with a predominant TNF-α production and low IL-10 secretion induced by two final CD4-predicted epitopes of F3, FRYPRPKHCHTQVA, and KFWCLVIDALKRIG (21). The FRYPRPKHCHTQVA epitope is also the more potent enhancer of the CD4^+^TNF-α^+^, -IFN-γ^+^, -TNF-α^+^IL-2^+^, -TNF-α^+^IFN-γ^+^, and -IFN-γ^+^IL-2^+^ T cell proportions, confirming its capability to raise a specific Th1 response. Additionally, the multifunctional IL-2^+^TNF-α^+^IFN-γ^+^-secreting CD4^+^ T-cells were raised only, in response to the FRYPRPKHCHTQVA and FMLQILDFYTKVYE (21).

NH36 is a strong phylogenetic marker of the genus *Leishmania*, which exhibits high identity of its amino acid sequence in all studied species of *Leishmania*. Hence, vaccination with NH36 in recombinant protein or DNA forms induced strong prophylactic effect against mice VL due to *L. (L.) donovani* and *L. (L.) infantum chagasi* (15, 16) and tegumentary leishmaniasis due to *L. (L.) amazonensis* (15, 21) and *L (L.) mexicana* (16).

However, recent advances in vaccinology demonstrate that vaccine-induced protection could be enhanced by the identification of the main domains or epitopes of the whole protein. In fact, vaccines that contain the short peptides, which represent the immunogenic epitopes, are able to optimize and even exceed the protective potential induced by the whole cognate protein (15, 21, 26, 49). These short peptide vaccines can also induce universal T cell responses, which are related to many human HLA-DR allotypes and to diverse mice strains (50, 51). This is the rationale of the development of T-epitope vaccines and it was also the guide for the development of the NH36 vaccines (15, 21, 26).

In fact, although 1 M of F3 (13,100 g/l) represents only 38% of 1 M of NH36 (34,240 g/l), its sequence is the most immunogenic in the NH36 molecule. The three CD4^+^ T cell epitopes of NH36: FMLQILDFYTKVYE (1,810.13 g/l), FRYPRPKHCHTQVA (1,740.01 g/l), and KFWCLVIDALKRIG (1,620 g/l) correspond to a 42 amino acid sequence totally located in F3. Taken together, 1 M of these 42 amino acids (5,180 g/l) constitutes 39% of the sequence of the F3 peptide (13,100 KDa), which is composed of the last 115 amino acids of NH36, but represents only 15% of the sequence of the whole NH36 protein (34,240 KDa), which is composed of 314 amino acids. Therefore, although the most potent epitopes are also present in NH36, in the F3 domain they are 2.6 times more concentrated. That is why the F3 vaccine exceeds the protective potential of the NH36 whole protein. The superiority of the F3 peptide domains over the NH36 vaccine in prophylaxis was previously observed in several parasitological and immunological variables before and it was calculated according to the following equation = (F3-NH36/F3) values × 100 = protective effect increment (15, 26). This is a concept related to the purification yield of an active peptide by biochemical techniques. As expected, using that calculation we found a 36% average stronger protective response induced by the F3 vaccine against mice infection by *L. (L.) infantum chagasi* (15) and a 40% enhanced response against challenge by *L. (L.) amazonensis* (26). Accordingly, in this investigation, the F3 vaccine promoted an increment of 80% in the expression of CCR7, of 42% in the migration of DCs to the wp, and of 54% in the reduction of the spleen parasite load after immunotherapy induced by the F3 vaccine.

Although it is true that F3 holds the most important epitopes of NH36, the adaptive immune response starts with the interaction of DCs with lymphocytes, and the reasons why the F3 vaccine induced a higher expression of CCR7 and stronger migration of DCs than NH36 are not yet, fully understood.

F3 epitopes not only interact more than the rest of the NH36 epitopes with the G-protein-coupled kinin receptors (BR2) (47), but they are also more expressed more by the MHC class II receptors (15, 21, 26) and induce a higher expression of CCR7. However, the CCR7 expression alone does not guarantee the correct DC migration toward CCL19 or CCL21 gradients (52). A second signal, mediated by prostaglandin E2 (PGE2), is responsible for DC migration. The PGE2–EP4 axis is involved not only in migration, but also in upregulation of co-stimulatory molecules and increased T cells activation (52), and thus is, crucial for the development of the immune response. In contrast, activation of the liver X receptor (LXR)α interferes with CCR7 expression and migration of DCs resulting in a reduced immune response. PGE2 was recently demonstrated to downregulate LXRα expression in *ex vivo* DCs, by enhancing CCR7 expression and migration of LXR-activated DCs (52). These facts indicate that the F3 peptide and its epitopes, more than the whole NH36 antigen, not only promote the upregulated expression of CCR7, but also probably the PGE2 signaling and stimulation of DCs, which triggers the Th1 immune response against *Leishmania (L.) infantum chagasi*. This hypothesis deserves further studies.

Our findings suggest that DCs can be used in efficacious antigen transportation in vaccination protocols against VL (41). Other infection models using *Mycobacterium tuberculosis* (53), *Chlamydia trachomatis* (54), and other infectious diseases and cancer (55) have shown that immunizations performed with DCs previously incubated with the antigen, as natural adjuvant, can achieve the generation of protective pathogen-specific T cell responses. Although the use of DCs in immunotherapeutic protocols has been considered a promising tool to induce effective immunity against VL (41), the limitations of using DC base therapeutic immunization and prevention have also been extensively discussed (56, 57).

The main contribution of our investigation is the description of the enhancement of CCR7 expression and the migrating DCs capabilities generated by the F3 vaccine. This vaccine property might be an important trigger of the Th1 response and of an immunotherapeutic effect against VL.

## Ethics Statement

All animals studies followed the guidelines set by the National Institutes of Health, USA, and the Institutional Animal Care and Use Committee approved the animal protocols (Comissão de Ética no Uso de Animais da Universidade Federal do Rio de Janeiro, CEUA protocol IMPPG040-07/16). All animal experimentation was performed in accordance with the terms of the Brazilian guidelines for the animal welfare regulations. Animals were kept at the Instituto de Microbiologia Paulo de Góes, da Universidade Federal do Rio de Janeiro (UFRJ) facilities, with controlled temperature, 12 h light/dark cycles and given water and feed *ad libitum*. We made all efforts in order to minimize animal suffering.

## Author Contributions

DN, AM, FA, and JM conducted the experiments. DN, AM, CF-d-L, LF-d-L, and FC acquired data. DN, FA, PL, CP-d-S analyzed data. CP-d-S, AM, and AMBM designed research studies. DN and CP-d-S wrote the manuscript. All authors have read and approved the final manuscript.

## Conflict of Interest Statement

DN and CP-d-S are the inventors of the patent file PI1015788-3 (INPI Brazil). AM, FA, FC, AMBM, JM, CF-d-L, PL, and LF-d-L, declare no conflict of interest.

## Supplementary Material

Figure S1 Schematic representation of dendritic cells (DCs) purification method and transwell experiment. DCs of mice vaccinated with F3 and challenged with *Leishmania (L.) infantum chagasi* migrate from the upper to the lower chamber of a transwell plate, in response to the CCL19 chemokine gradient. In contrast, DCs from unvaccinated infected mice are unresponsive to CCL19 and do not migrate.

Figure S2 Strategy for the analysis of multifunctional T cell response using a four-color flow cytometry panel to simultaneously analyze multiple cytokines at the single-cell level in splenocytes cultures. After single cells selection (FSC-A × FSC-H), lymphocytes from a representative mouse immunized with the F3 vaccine were selected according to a FSC-A versus SSC-A dot plot, followed by CD4^+^ **(A)** or CD8^+^ **(B)** gating. Afterward, CD4^+^ or CD8^+^ T-cell phenotypes were plotted against each cytokine individually: interferon-γ (IFN-γ), tumor necrosis factor-α (TNF-α), and interleukin (IL)-2. Boolean gating was performed to generate the frequencies of the possible seven combinations of cytokine-producing CD4^+^ or CD8^+^ cells using FlowJo V10 software. In this demonstrative figure, we show the results obtained with splenocytes from an animal immunized with the F3 vaccine, 15 days after infection, stimulated “*in vitro*” with the NH36 recombinant protein.

Figure S3 Cytokines expressed by CD4^+^ T lymphocytes in response to *Leishmania (L.) infantum chagasi* lysate. Effect of the F3 and NH36-vaccines on the frequencies of CD4^+^IL-2^+^ **(A)**, TNF-α^+^ **(D)**, IFN-γ^+^ **(G)**, TNF-α^+^IL-2^+^ **(B)**, TNF-α^+^IFN-γ^+^ **(E)**, IL-2^+^IFN-γ^+^ **(H)**, and IL-2^+^TNF-α^+^IFN-γ^+^-secreting T cells **(C)** in response to the promastigote lysate, on day 15 and 28 post challenge. The total CD4^+^ T cell frequencies are also represented **(F)**. Bars represent the mean + SE values of two-independent experiments (*n* = 5 mice per treatment in each experiment). Asterisks and horizontal lines show significant differences between treatments as disclosed by Mann–Whitney non-parametrical test.

Figure S4 Cytokines expressed by CD8^+^ T lymphocytes to *Leishmania (L.) infantum chagasi* lysate. Effect of the F3 and NH36-vaccines on the frequencies of CD8^+^IL-2^+^ **(A)**, TNF-α^+^ **(D)**, IFN-γ^+^ **(G)**, TNF-α^+^IL-2^+^ **(B)**, TNF-α^+^IFN-γ^+^ **(E)**, IL-2^+^IFN-γ^+^ **(H)**, and IL-2^+^TNF-α^+^IFN-γ^+^-secreting T cells **(C)** in response to the promastigote lysate, on day 15 and 28 post challenge. The total CD8^+^ T cell frequencies are also represented **(F)**. Bars represent the mean + SE values of two-independent experiments (*n* = 5 mice per treatment in each experiment). Asterisks and horizontal lines show significant differences between treatments as disclosed by Mann–Whitney non-parametrical test.
