# Supplementary figures and images for: NH36 and F3 Antigen-Primed Dendritic Cells Show Preserved Migrating Capabilities and CCR7 Expression and F3 Is Effective in Immunotherapy of Visceral Leishmaniasis
Source: Front Immunol. 2018 May 7;9:967. doi: 10.3389/fimmu.2018.00967 (PMC5949526; doi:10.3389/fimmu.2018.00967)

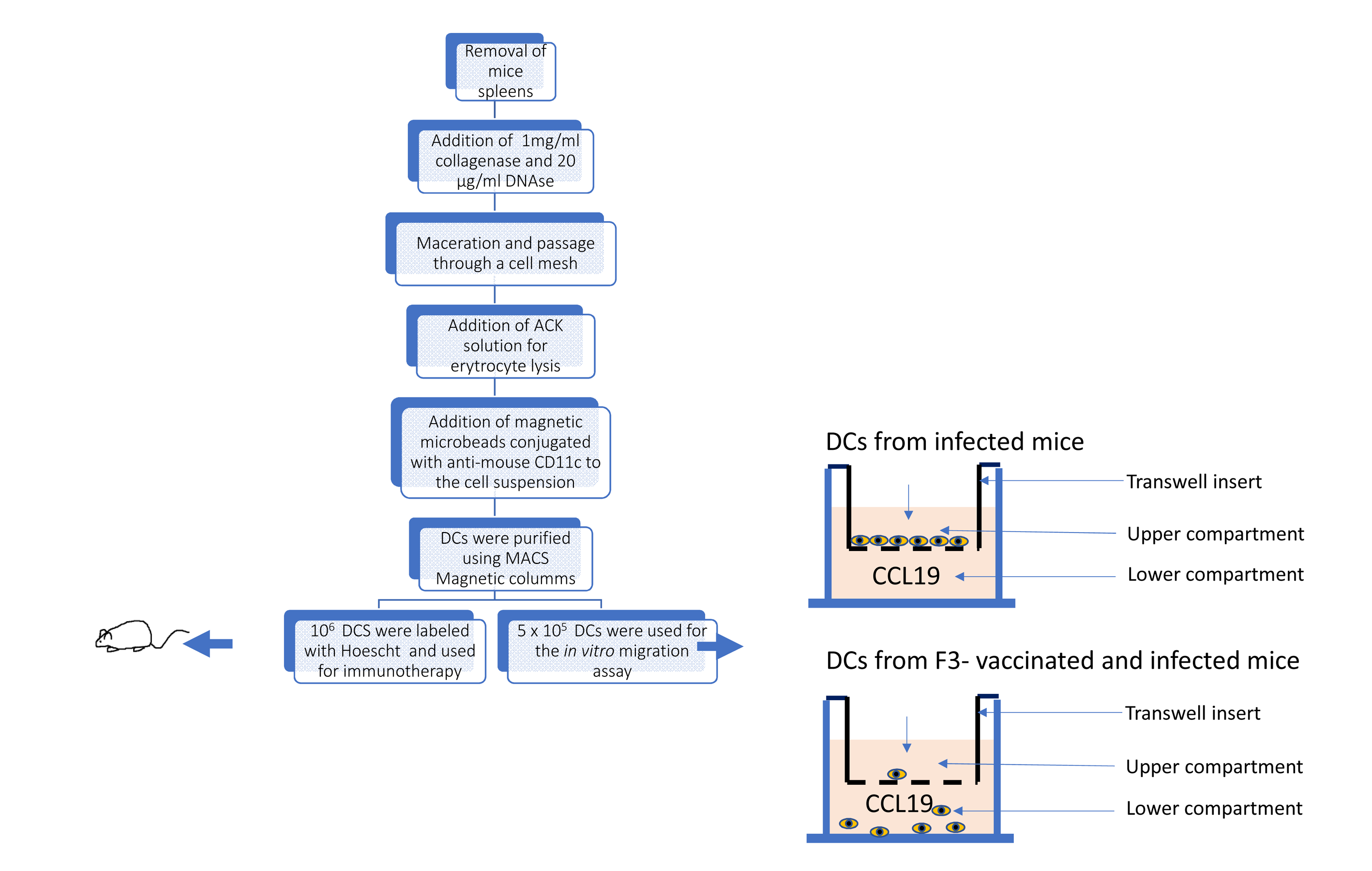

Supplement: Figure S1 — Schematic representation of dendritic cells (DCs) purification method and transwell experiment. DCs of mice vaccinated with F3 and challenged with Leishmania (L.) infantum chagasi migrate from the upper to the lower chamber of a transwell plate, in response to the CCL19 chemokine gradient. In contrast, DCs from unvaccinated infected mice are unresponsive to CCL19 and do not migrate. [file Image_1.tif]

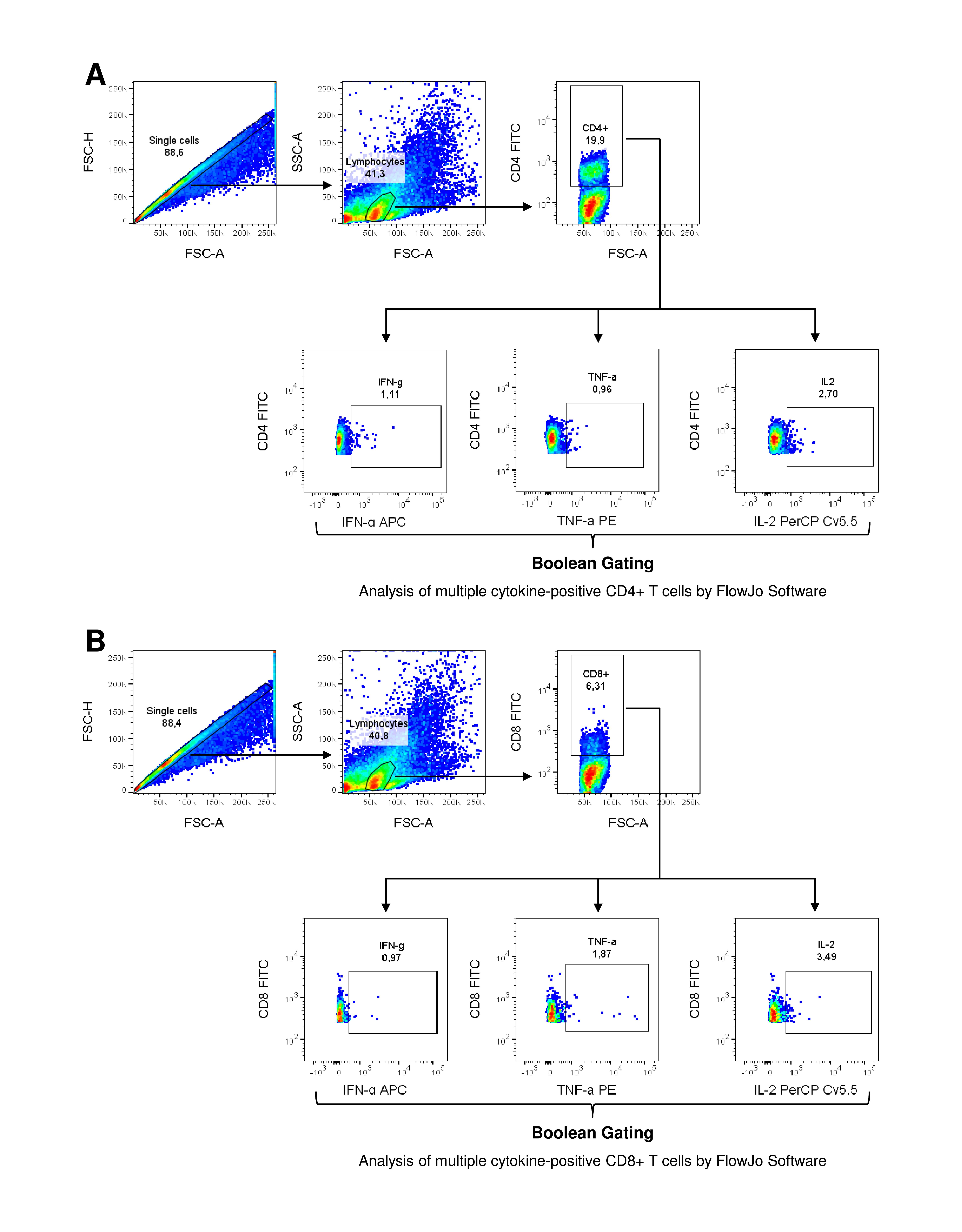

Supplement: Figure S2 — Strategy for the analysis of multifunctional T cell response using a four-color flow cytometry panel to simultaneously analyze multiple cytokines at the single-cell level in splenocytes cultures. After single cells selection (FSC-A × FSC-H), lymphocytes from a representative mouse immunized with the F3 vaccine were selected according to a FSC-A versus SSC-A dot plot, followed by CD4+ (A) or CD8+ (B) gating. Afterward, CD4+ or CD8+ T-cell phenotypes were plotted against each cytokine individually: interferon-γ (IFN-γ), tumor necrosis factor-α (TNF-α), and interleukin (IL)-2. Boolean gating was performed to generate the frequencies of the possible seven combinations of cytokine-producing CD4+ or CD8+ cells using FlowJo V10 software. In this demonstrative figure, we show the results obtained with splenocytes from an animal immunized with the F3 vaccine, 15 days after infection, stimulated “in vitro” with the NH36 recombinant protein. [file Image_2.tif]

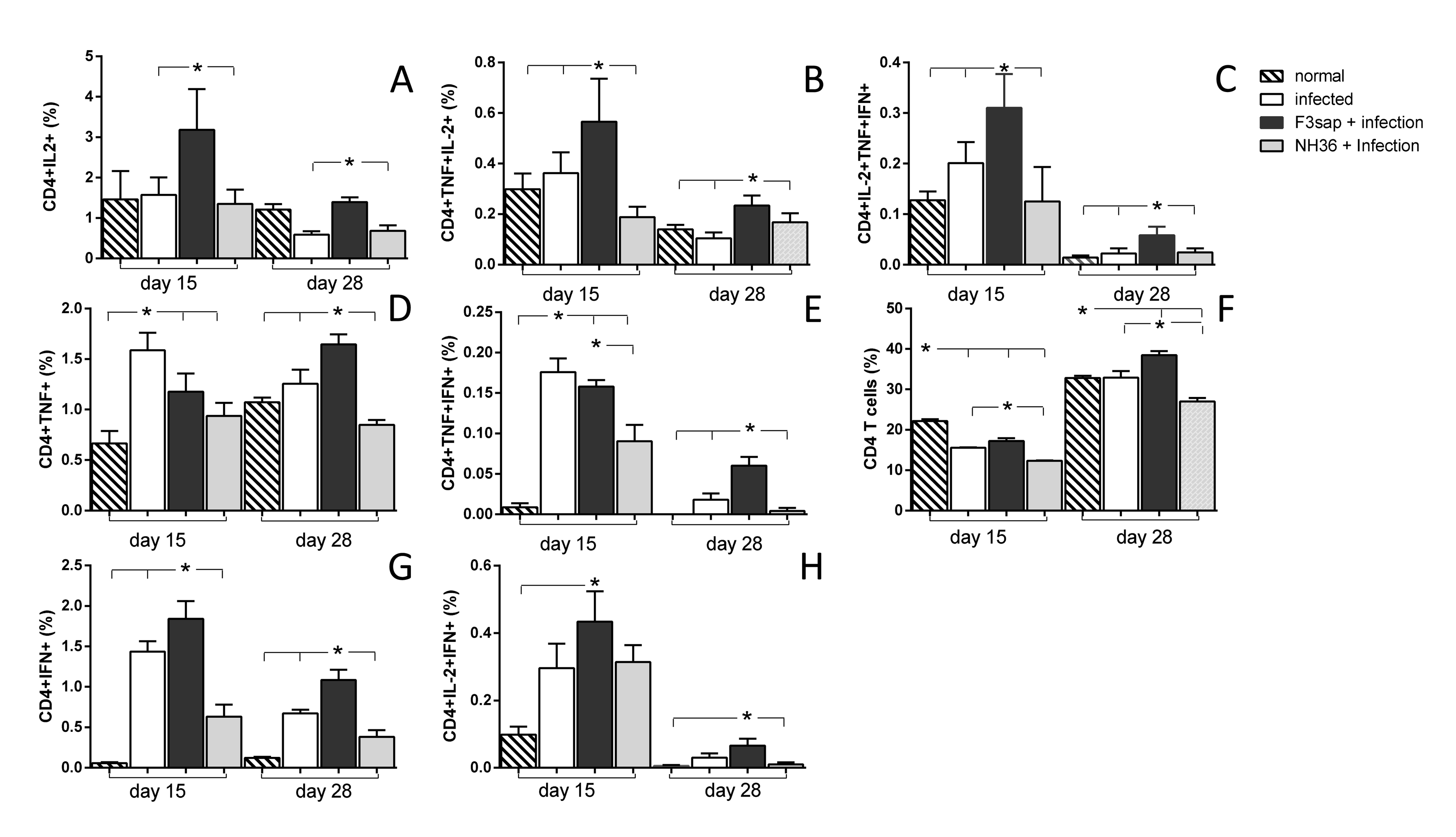

Supplement: Figure S3 — Cytokines expressed by CD4+ T lymphocytes in response to Leishmania (L.) infantum chagasi lysate. Effect of the F3 and NH36-vaccines on the frequencies of CD4+IL-2+ (A), TNF-α+ (D), IFN-γ+ (G), TNF-α+IL-2+ (B), TNF-α+IFN-γ+ (E), IL-2+IFN-γ+ (H), and IL-2+TNF-α+IFN-γ+-secreting T cells (C) in response to the promastigote lysate, on day 15 and 28 post challenge. The total CD4+ T cell frequencies are also represented (F). Bars represent the mean + SE values of two-independent experiments (n = 5 mice per treatment in each experiment). Asterisks and horizontal lines show significant differences between treatments as disclosed by Mann–Whitney non-parametrical test. [file Image_3.jpg]

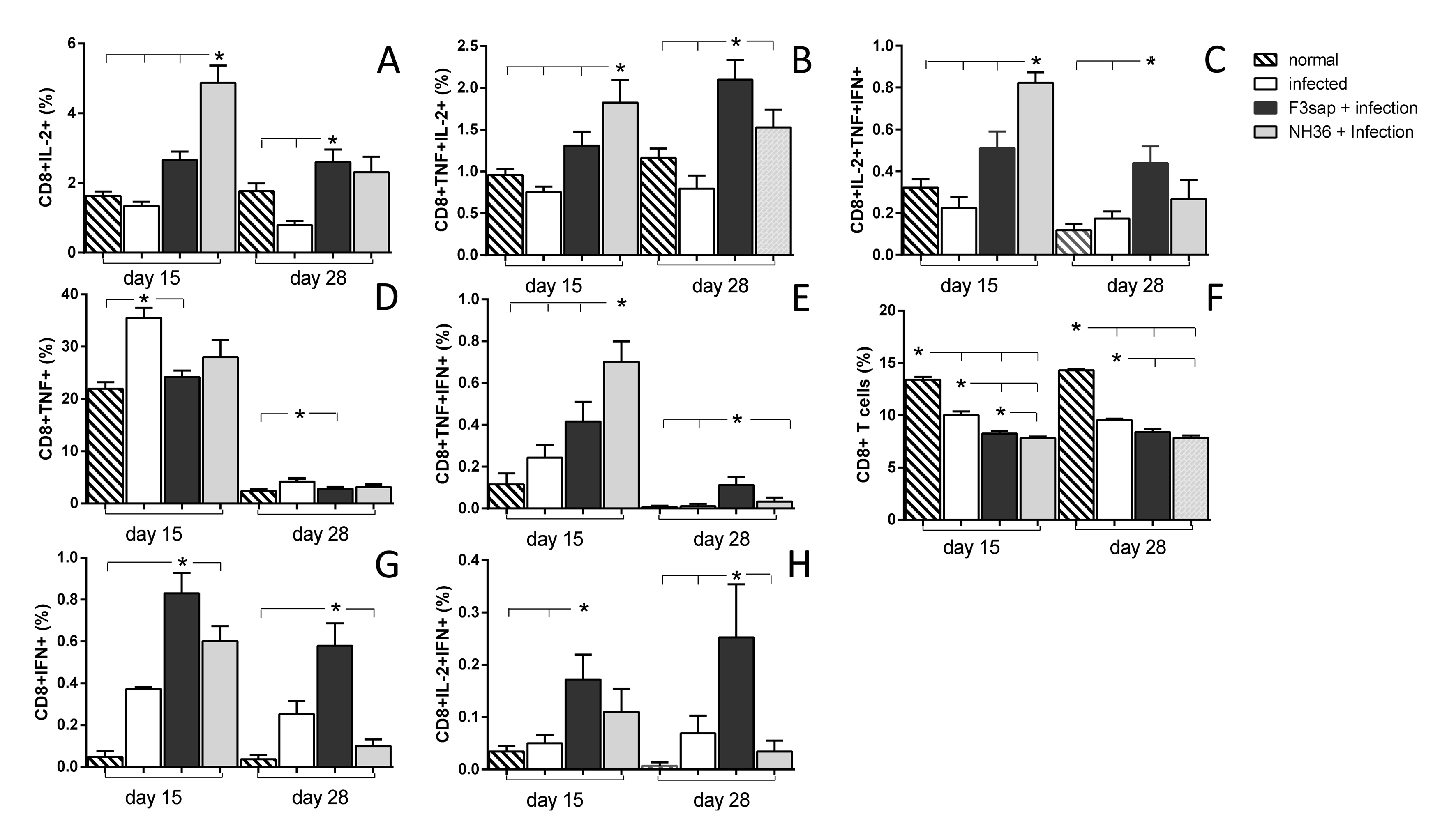

Supplement: Figure S4 — Cytokines expressed by CD8+ T lymphocytes to Leishmania (L.) infantum chagasi lysate. Effect of the F3 and NH36-vaccines on the frequencies of CD8+IL-2+ (A), TNF-α+ (D), IFN-γ+ (G), TNF-α+IL-2+ (B), TNF-α+IFN-γ+ (E), IL-2+IFN-γ+ (H), and IL-2+TNF-α+IFN-γ+-secreting T cells (C) in response to the promastigote lysate, on day 15 and 28 post challenge. The total CD8+ T cell frequencies are also represented (F). Bars represent the mean + SE values of two-independent experiments (n = 5 mice per treatment in each experiment). Asterisks and horizontal lines show significant differences between treatments as disclosed by Mann–Whitney non-parametrical test. [file Image_4.jpg]
